# HUWE1‐Mediated Degradation of MUTYH Facilitates DNA Damage and Mitochondrial Dysfunction to Promote Acute Kidney Injury

**DOI:** 10.1002/advs.202412250

**Published:** 2025-02-08

**Authors:** Yunwen Yang, Peipei Wang, Kaiqian Zhou, Wen Zhang, Suwen Liu, Jing Ouyang, Mi Bai, Guixia Ding, Songming Huang, Zhanjun Jia, Aihua Zhang

**Affiliations:** ^1^ Department of Nephrology Children's Hospital of Nanjing Medical University 72 Guangzhou Road Nanjing 210008 P. R. China; ^2^ Nanjing Key Laboratory of Pediatrics Children's Hospital of Nanjing Medical University Nanjing 210008 P. R. China; ^3^ Jiangsu Key Laboratory of Early Development and Chronic Diseases Prevention in Children Nanjing Medical University Nanjing 210029 P. R. China; ^4^ Department of Nephrology Affiliated Hospital of Integrated Traditional Chinese and Western Medicine Nanjing University of Chinese Medicine Nanjing 210028 P. R. China; ^5^ Department of Pediatrics Shandong Provincial Hospital Affiliated to Shandong First Medical University Jinan 250021 P. R. China

**Keywords:** acute kidney injury, base excision repair, DNA damage, HUWE1, MUTYH

## Abstract

The role of MUTYH, a DNA repair glycosylase in the pathogenesis of acute kidney injury (AKI) is unclear. In this study, it is found that MUTYH protein levels are significantly decreased in the kidneys of cisplatin‐ or folic acid (FA)‐induced mouse AKI models and patients with AKI. MUTYH deficiency aggravates renal dysfunction and tubular injury following cisplatin and FA treatment, along with the accumulation of 7, 8‐dihydro‐8‐oxoguanine (8‐oxoG) and impairs mitochondrial function. Importantly, the overexpression of type 2 MUTYH (nuclear) significantly ameliorates cisplatin‐induced apoptosis, oxidative stress, mitochondrial dysfunction, and DNA damage in vivo and in vitro. In contrast, overexpression of type 1 MUTYH (mitochondrial) shows a marginal effect against cisplatin‐induced injury, indicating the chief role of type 2 MUTYH in antagonizing AKI. Interestingly, the results also indicate that the upregulation of the E3 ligase HUWE1 causes the ubiquitination and degradation of MUTYH in tubular epithelial cells. HUWE1 knockout or treatment with the HUWE1 inhibitor BI8622 significantly protect against cisplatin‐induced AKI. Taken together, these results suggest that the ubiquitin E3 ligase HUWE1‐mediates ubiquitination and degradation of MUTYH can aggravate DNA damage in the nucleus and mitochondria and promote AKI. Targeting the HUWE1/MUTYH pathway may be a potential strategy for AKI treatment.

## Graphical abstract

MutY homolog (MUTYH) protein levels are significantly decreased in the kidneys of acute kidney injury (AKI) patients. The upregulation of the E3 ligase HUWE1 mediates the degradation of MUTYH in renal tubular epithelial cells, exacerbating DNA damage in both the nucleus and mitochondria, thereby promoting AKI. Targeting the HUWE1/MUTYH pathway may be a potential strategy for AKI treatment.

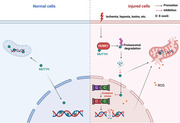

## 1 Introduction

Acute kidney injury (AKI) is a clinical condition characterized by an acute and usually transient decrease in renal function. Clinical studies show that AKI is a life‐threatening condition with high morbidity and mortality. AKI occurs in ≈13% of hospitalized patients, contributes to ≈1.7 million deaths worldwide each year, and predisposes patients to chronic kidney disease (CKD) and end‐stage renal disorders (ESRD), which affect ≈10% of the world's population.^[^ ^1^ ^]^ Renal proximal tubule epithelial cells, the main component cells of the renal parenchyma, are the primary targets of a variety of ischemic or toxic insults.^[^ ^2^ ^]^ Renal tubular cell death is an early event in AKI, followed by the dedifferentiation, proliferation, and regeneration of renal tubules. This process is accompanied by cytokines and damage‐associated molecular patterns (DAMPs) in injured cells.^[^ ^3^ ^]^ Over the past decades, the nephrology community has explored the molecular mechanisms underlying AKI and has found that multiple cell death pathways are involved in AKI.^[^ ^4^ ^]^ However, no satisfactory treatment for AKI in the clinic has been established, and the precise molecular mechanisms remain elusive.

Chemotherapy drugs, UV light, and endogenous stressors, such as reactive oxygen species (ROS) can induce cellular DNA damage.^[^ ^5^ ^]^ DNA damage includes single‐ and double‐strand breaks, base loss, and mismatched base pairs.^[^ ^5^ ^]^ DNA damage is a feature of many types of kidney injury induced by ischemia or toxic insults, such as cisplatin.^[^ ^6^ ^]^ When cellular DNA damage occurs, a sensitive and complex DNA damage response (DDR) is activated to repair the damage.^[^ ^6d^ ^]^ DNA strand breaks in cells are detected by one or more of the major DDR sensor kinases, including ataxia telangiectasia mutated (ATM), ATM‐ and Rad3‐related (ATR), and DNA‐dependent protein kinase (DNA‐PK). The activation of these sensor kinases activates checkpoint kinases 1 and 2 (Chk1 and Chk2), which regulate cyclin‐dependent kinase 1 (CDK1) and CDK2, resulting in G1/S and G2/M arrest for DNA repair.^[^ ^7^ ^]^ However, incomplete DNA repair leads to cell dysfunction, cell cycle arrest, senescence, and apoptosis.^[^ ^8^ ^]^ Previous studies have demonstrated that the DDR is activated and protects against kidney damage in cisplatin‐ or ischemia‐induced AKI.^[^ ^6^, ^8^ ^]^ In contrast, DNA bases are susceptible to oxidation by ROS, and guanine, with a low redox potential, is particularly vulnerable, resulting in the generation of 7,8‐dihydro‐8‐oxoguanine (8‐oxoG), which is one of the best‐characterized oxidative DNA lesions.^[^ ^9^ ^]^ An 8‐oxoG lesion is highly mutagenic, as it pairs with adenine (A), leading to A: 8‐oxoG mispairing during DNA replication. Failure to remove 8‐oxoG before DNA replication results in G: C to T: A transversion mutations.^[^ ^10^ ^]^ Previous studies have shown that mismatched base pairs are repaired via the base excision repair (BER) pathway.^[^ ^10^, ^11^ ^]^ Although 8‐oxoG has been detected in kidneys under several conditions,^[^ ^12^ ^]^ the role of the BER pathway in AKI is unknown.

The DNA repair glycosylase OGG1 and the mammalian homolog of *Escherichia coli* MutY (MUTYH) constitute the BER pathway for 8‐oxoG repair in eukaryotes.^[^ ^11b^ ^]^ OGG1 removes 8‐oxoG from 8‐oxoG: C base pairs, and MUTYH recognizes and removes A from 8‐oxoG: A mispairs, thus preventing the potential of G: C to T: A transversion mutations from occurring during DNA replication, which is important in protecting against oxidative DNA damage.^[^ ^10^, ^13^ ^]^ Loss or mutation of MUTYH and accumulation of oxidative DNA damage have been reported in patients with a hereditary colorectal cancer predisposition syndrome called MUTYH‐associated polyposis (MAP).^[^ ^14^ ^]^ Previous studies have shown that MUTYH knockout mice have an increase in base mutation frequency and a higher incidence of tumor formation compared to wild‐type (WT) mice.^[^ ^15^ ^]^ Moreover, MUTYH deficiency exacerbates oxidative DNA damage when mice are exposed to oxidants.^[^ ^16^ ^]^ Thus, MUTYH plays an important role in the repair of oxidative DNA damage and has also been reported in other diseases, including neurological disorders.^[^ ^17^ ^]^ A recent study showed that MUTYH plays an important role in maintaining mitochondrial homeostasis upon exposure to oxidants.^[^ ^16^ ^]^ The disruption of mitochondrial homeostasis, also known as mitochondrial dysfunction, is a pathogenic factor in AKI.^[^ ^18^ ^]^ However, the effects of MUTYH deficiency on the BER system have not been investigated in renal disease.

HECT, UBA, and WWE domain‐containing protein 1 (HUWE1), also known as ARF‐BP1, HectH9, or MULE, is a critical HECT domain E3 ubiquitin ligase in eukaryotes.^[^ ^19^ ^]^ HUWE1 is evolutionarily conserved and plays significant roles in various biological processes, including apoptosis,^[^ ^19^ ^]^ DNA damage repair,^[^ ^20^ ^]^ proliferation,^[^ ^21^ ^]^ and so on. It regulates the protein levels of numerous diverse substrates, such as P53, c‐MYC,^[^ ^21^ ^]^ and MCL‐1,^[^ ^19^ ^]^ to facilitate their functions. Although HUWE1 has been shown to mediate the turnover of MUTYH in tumor cells,^[^ ^22^ ^]^ its role in AKI remains unknown.

In this study, the expression of MUTYH was examined in two models of AKI, including a cisplatin‐ and folic acid (FA)‐induced mouse model, as well as in renal biopsy samples from patients with AKI. Using MUTYH knockout mice and proximal tubular cells with MUTYH overexpression in vivo and in vitro, we demonstrated that MUTYH protects against AKI by ameliorating oxidative DNA damage and mitochondrial dysfunction. Our findings suggest a potential strategy for AKI treatment.

## 2 Results

### 2.1 MUTYH was Downregulated in the Kidneys of Patients with AKI and AKI Mice

**Figure 1:**
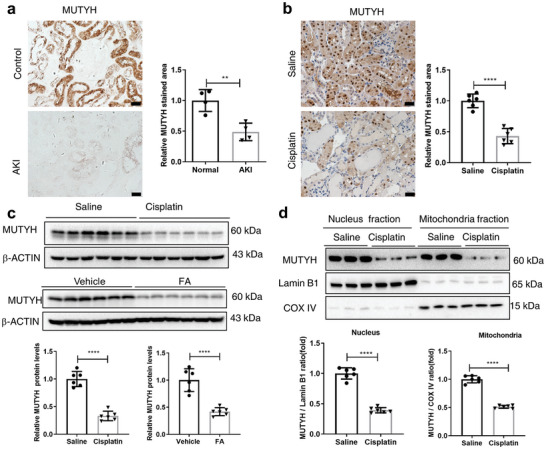
MUTYH is downregulated in the kidneys of patients with AKI and cisplatin‐treated mice. a) Immunohistochemical staining for MUTYH in the kidneys of patients with AKI (*n* = 4) and paracarcinoma (*n* = 4) (magnification: ×400, scale bar: 20 µm). Quantification of immunohistochemical staining for MUTYH in patients with AKI analyzed by Image J is shown on the right. Data are presented as mean ± S.D. from five random fields of each kidney. b) Immunohistochemical staining for MUTYH in kidneys of cisplatin‐induced mice (cisplatin treated for 72 h). c) Western blot analysis of MUTYH protein levels in the kidneys of cisplatin‐ or FA‐induced AKI mice (72 h). The graph shows the densitometry analysis results. Data are presented as mean ± S.D. from six mice from each group. ***P < 0.001 (*t*‐test). d) The protein levels of nuclear and mitochondrial MUTYH in in the kidneys of cisplatin‐treated (72 h) mice were analyzed by fraction Western blot. Mean ± S.D. for six animals per group (*n* = 6), ***P < 0.001 (*t*‐test).

To explore the association between MUTYH and human AKI, immunohistochemistry (IHC) staining was performed to analyze the expression levels of MUTYH in kidney biopsies from four patients with various forms of AKI, while paracarcinoma kidney samples from four patients who underwent renal carcinoma resection were used as controls. The results showed that the expression levels of MUTYH were significantly downregulated compared to controls, and MUTYH was mainly expressed in tubular cells (**Figure** **1**a; and Figure S1a, Supporting Information). MUTYH is a DNA repair enzyme that plays a key role in BER, and it is reasonable to speculate that MUTYH plays an important role in cisplatin‐induced AKI, as DNA damage is a key mechanism of action of cisplatin. To investigate whether MUTYH is involved in cisplatin‐induced AKI, a mouse model of cisplatin‐induced AKI was constructed. IHC (Figure 1b) and immunofluorescent (IF) (Figure S1b, Supporting Information) staining showed MUTYH was mainly expressed in renal tubule cells and decreased significantly after cisplatin treatment in kidneys of mice. Western blot showed that the protein levels of MUTYH were significantly downregulated in the kidneys of cisplatin‐treated mice compared to those of the control group (Figure 1c; and Figure S1c, Supporting Information). Moreover, MUTYH protein levels were also significantly downregulated in the kidneys of mice with FA‐induced AKI (Figure 1c). The MUTYH expression in both the nucleus and mitochondria was also examined. Western blot revealed that MUTYH protein levels decreased significantly after cisplatin treatment in both the nucleus and mitochondria isolated from the renal cortex (Figure 1d). Overall, these results suggest that the downregulation of MUTYH protein levels may play an important role in AKI.

### 2.2 MUTYH Deficiency Aggravated Cisplatin‐Induced AKI, DNA Damage, and Mitochondrial Dysfunction

**Figure 2:**
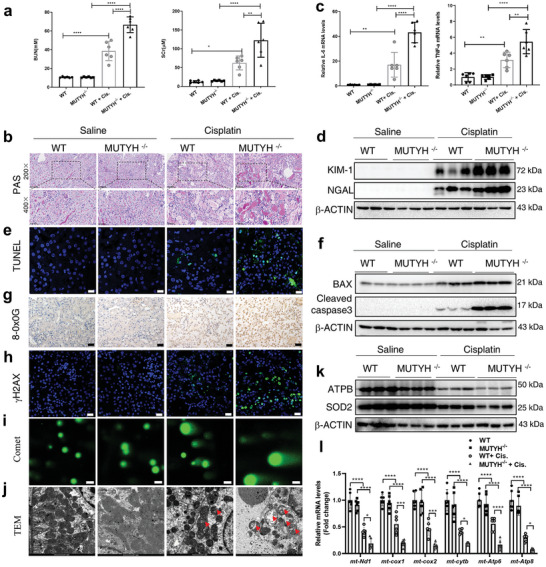
MUTYH knockout aggravates cisplatin‐induced AKI, DNA damage, and mitochondrial dysfunction in vivo. a) Scr and BUN levels in WT and MUTYH^−/−^ mice treated with cisplatin for 72 h. b) PAS staining (magnification: ×200 and 400) of kidneys from WT and MUTYH^−/−^ mice treated with or without cisplatin for 72 h. c) qRT‐PCR was performed to analyze the renal IL‐6 and TNF‐α mRNA levels. d) Western blot analysis of KIM‐1 and NGAL protein levels in the kidneys of cisplatin‐treated WT and MUTYH^−/−^ mice. e) TUNEL staining of the kidneys of cisplatin‐treated WT and MUTYH^−/−^ mice. f) The protein levels of Bax and cleaved caspase‐3 in the kidneys of cisplatin‐treated WT and MUTYH^−/−^ mice were analyzed by Western blot. g) Immunohistochemical staining for 8‐oxoG (original magnification: 400×; scale bar: 20 µm). h) Immunofluorescent staining for γH2AX in the kidneys of WT and MUTYH^−/−^ mice treated with cisplatin for 72 h. (green: γH2AX, blue, DAPI; magnification: 400×, scale bar: 20  µm). i) Comet tail moment in the kidneys of WT and MUTYH^−/−^ mice treated with cisplatin for 72 h. (green: DNA; magnification: 200×, scale bar: 50  µm). j) Electron microscopy images and quantification of damaged mitochondria in renal tubular cells of kidneys treated with cisplatin for 72 h (scale bar: 1  µm; red arrow: damaged mitochondria). k) Western blot analysis of SOD2 and ATPB protein levels. The genes encoded by the mitochondrial genome were analyzed using qRT‐PCR l). The quantitative results are shown as mean ± S.D. for six mice per group (*n* = 6). Cis: cisplatin, *P < 0.05, **P < 0.01, ***P < 0.001 (one‐ or two‐way ANOVA).

To elucidate whether the downregulation of MUTYH promotes cisplatin‐induced AKI, MUTYH knockout (MUTYH^−/−^) mice were bred and used to construct a cisplatin‐induced AKI mouse model. Genotyping (Figure S2a, Supporting Information), Western blot (Figure S2b, Supporting Information), and quantitative real‐time PCR (qRT‐PCR) (Figure S2c, Supporting Information) confirmed the successful construction of MUTYH knockout mice. Changes in body weight and food intake were not significantly different between cisplatin‐treated WT and MUTYH^−/−^ mice (data not shown). As shown in **Figure** **2**a, higher levels of BUN and Scr indicated that MUTYH deficiency aggravated cisplatin‐induced renal dysfunction compared to that of WT controls. Histological analysis by periodic acid‐Schiff (PAS) staining showed that compared with WT mice, MUTYH^−/−^ mice showed markedly aggravated tubular injury (Figure 2b; and Figure S2d, Supporting Information). The inflammatory response is another significant pathological feature of cisplatin‐induced nephrotoxicity, and our results showed that MUTYH deficiency aggravated cisplatin‐induced renal inflammation compared to that in WT mice (Figure 2c; and Figure S2e, Supporting Information). Additionally, the protein levels of renal tubular injury markers, including neutrophil gelatinase associated lipocalin (NGAL) and kidney injury molecule 1 (KIM‐1), were analyzed using Western blot. The results showed that the protein levels of NGAL and KIM‐1 were much higher in the kidneys of MUTYH^−/−^ mice than in the kidneys of WT mice after cisplatin treatment (Figure 2d; and Figure S2f, Supporting Information). TUNEL staining and western blot were performed to analyze cisplatin‐induced apoptosis, and the results showed that the number of TUNEL‐positive cells (Figure 2e; and Figure S2g, Supporting Information) and the levels of cleaved caspase‐3 and Bax (Figure 2f; and Figure S2h, Supporting Information) in the kidneys of MUTYH^−/−^ mice were higher than those in the kidneys of WT mice after cisplatin treatment.

MUTYH is a DNA damage‐specific glycosylase that initiates BER by recognizing and removing 8‐oxoG and its paired adenine, which plays an important role in preventing DNA damage. IHC showed that the levels of 8‐oxoG in the kidneys of mice increased significantly after 72 h of cisplatin treatment. However, MUTYH deficiency aggravated the cisplatin‐induced accumulation of 8‐oxoG (Figure 2g; and Figure S2i,j, Supporting Information). Additionally, the levels of 8‐oxoG in nDNA or Mitochondrial DNA (mtDNA) were also analyzed separately, our results showed MUTYH deficiency aggravated the cisplatin‐induced accumulation of 8‐oxoG both in nDNA and mtDNA (Figure S2k, Supporting Information). Accumulation of 8‐oxoG exacerbated DNA damage and oxidative stress. IF showed that MUTYH deficiency resulted in higher levels of γH2AX, which has been widely used as a sensitive marker for DNA DSBs^[^ ^23^ ^]^ in the kidneys after cisplatin treatment compared to that of WT controls (Figure 2h; and Figure S2l, Supporting Information). Additionally, comet assays showed that MUTYH deficiency aggravated cisplatin‐induced DNA strand breaks (Figure 2i; and Figure S2m, Supporting Information). MUTYH is a BER protein that is important for nDNA and mtDNA damage repair. mtDNA stability is necessary to maintain mitochondrial function. Therefore, we examined whether MUTYH deficiency aggravated cisplatin‐induced mitochondrial damage. Electron microscopy images revealed that the number of damaged mitochondria with swollen and disrupted cristae in the renal tubular cells of cisplatin‐treated MUTYH knockout mice was markedly higher than that in their WT littermates (Figure 2j; and Figure S2n, Supporting Information). Western blot showed that the protein levels of mitochondrial SOD (SOD2) and ATPB were lower after cisplatin treatment in MUTYH knockout mice than in their WT littermates (Figure 2k; and Figure S2o, Supporting Information). Additionally, the mitochondrial copy number and mRNA levels of mtDNA‐encoded genes, including *mt‐nd1, mt‐cox1, mt‐cox2, mt‐cytb, mt‐ATP6*, and *mt‐ATP8*, were analyzed using qRT‐PCR. The results showed that MUTYH knockout aggravated the cisplatin‐induced downregulation of the mitochondrial copy number (Figure S2p, Supporting Information) and expression of mtDNA‐encoded genes (Figure 2l). These results indicate that MUTYH deficiency aggravated cisplatin‐induced nephrotoxicity, DNA damage, and mitochondrial dysfunction in mice.

### 2.3 MUTYH Deficiency Aggravated Cisplatin‐Induced Apoptosis, DNA Damage, and Mitochondrial Dysfunction In Vitro

**Figure 3:**
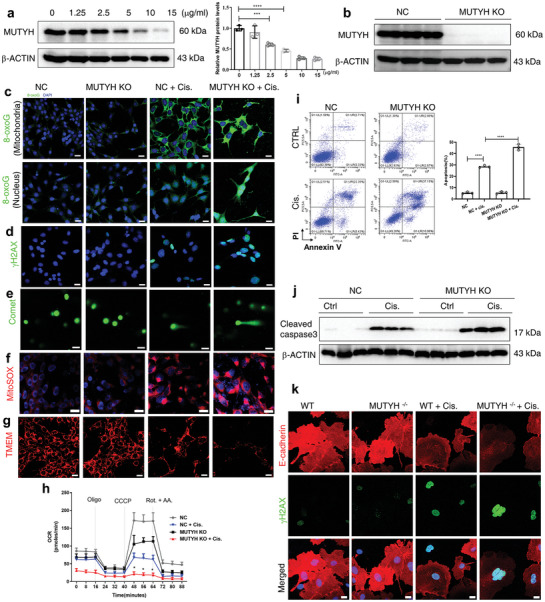
MUTYH knockout aggravates cisplatin induced apoptosis, DNA damage, and mitochondrial dysfunction in vitro. a) Western blot and quantification of MUTYH protein levels in mPTCs treated with different concentrations of cisplatin for 24 h. b) The protein levels of MUTYH in NC or MUTYH KO mPTCs were analyzed by Western blot. c) Immunofluorescent images of 8‐oxoG in mitochondria (upper) or nucleus (down) induced by cisplatin in NC or MUTYH KO mPTCs (scale bar: 20 µm, green: 8‐oxoG, blue: DAPI). d) Immunofluorescent images of γH2AX induced by cisplatin in NC or MUTYH KO mPTCs (scale bar: 20 µm, green: γH2AX, blue: DAPI). e) Cisplatin‐induced comet tail moment in NC or MUTYH KO mPTCs (scale bar: 50 µm, green: DNA). f) Fluorescent staining for MitoSOX in NC or MUTYH KO mPTCs treated with cisplatin for 24 h (scale bar: 20 µm, red: MitoSOX, blue: DAPI). g) Fluorescent images of mitochondrial membrane potential (TMRM) in NC or MUTYH KO mPTCs treated with cisplatin for 24 h (scale bar: 20 µm, red: TMRM, blue: DAPI). h) Oxygen consumption rate (OCR) in NC or MUTYH KO mPTCs measured using a Seahorse 96 XF‐analyzer. i) Flow cytometry analysis of annexin V and PI staining and quantification of cisplatin‐induced apoptosis. j) The protein levels of cleaved caspase3 in NC or MUTYH KO mPTCs were analyzed by Western blot. k) Immunofluorescent images of E‐cadherin and γH2AX in WT or MUTYH KO primary mPTCs treated with cisplatin for 24 h (green: γH2AX, red: E‐cadherin, blue: DAPI; magnification: 400×, scale bar: 20  µm). Cell experiments were performed three times (*n* = 3), and data are expressed as mean ± S.D. NC: negative control, KO: knockout, Cis: cisplatin; ***P < 0.001, **P < 0.01, *P < 0.05 (two‐way ANOVA).

We further examined the role of MUTYH in cisplatin‐induced nephrotoxicity using mPTCs in vitro. Consistent with the results of animal studies, cisplatin treatment downregulated MUTYH protein levels in a dose‐dependent and time‐dependent manner (**Figure** **3**a; and Figure S3a, Supporting Information). To elucidate the role of MUTYH in mPTCs, MUTYH‐deficient mPTCs (MUTYH^−/−^) were constructed using the CRISPR/cas9 method, and qRT‐PCR (Figure S3b, Supporting Information) and western blot (Figure 3b; and Figure S3c, Supporting Information) were performed to confirm the successful construction of MUTYH^−/−^ mPTCs. Consistent with the in vivo results, MUTYH deficiency exacerbated cisplatin‐induced DNA damage, as evidenced by increased levels of 8‐oxoG in both nDNA and mtDNA (Figure 3c; and Figure S3d, Supporting Information), along with heightened levels of γH2AX (Figure 3d; and Figure S3e, Supporting Information) and comet tail moment (Figure 3e; and Figure S3f, Supporting Information). Next, we examined the effects of MUTYH deficiency on mitochondrial ROS production, mitochondrial membrane potential, and oxygen consumption rate (OCR) in cisplatin‐treated mPTCs. The results showed that MUTYH deficiency aggravated cisplatin‐induced ROS production (Figure 3f; and Figure S3g,h, Supporting Information), and MMP (Figure 3g; and Figure S3I, Supporting Information) and OCR (Figure 3h) were decreased compared to control cells. Additionally, MUTYH deficiency aggravated cisplatin‐induced cell death in mPTCs, as evidenced by increased apoptosis and cleaved caspase‐3 protein levels (Figure 3i,j; and Figure S3j, Supporting Information). Moreover, primary renal tubular cells were isolated form MUTYH^−/−^ and WT mice. Our results showed that MUTYH deficiency aggravated the cisplatin‐induced DNA damage (Figure 3k; and Figure S3k, Supporting Information) in primary renal tubular cells. These results show that MUTYH deficiency aggravated cisplatin‐induced apoptosis, DNA damage, and mitochondrial dysfunction in vitro.

### 2.4 Overexpression of Type 2 MUTYH Ameliorated AKI and Cisplatin‐Induced DNA Damage

**Figure 4:**
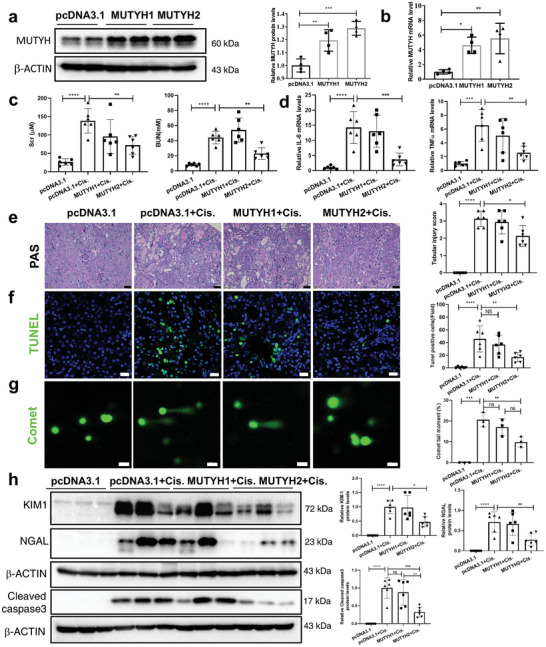
Overexpression of type 2 MUTYH ameliorates cisplatin‐induced AKI and DNA damage. a) Western blot analysis of MUTYH protein levels in the kidneys 36 h after injecting type 1 or 2 human MUTYH and vector plasmids. b) qRT‐PCR analysis of MUTYH mRNA levels in the kidneys 36 h after injecting plasmids. Mean ± S.D. of four animals per group. c) BUN and Scr levels in control and MUTYH overexpression mice (6 mice of each group) treated with cisplatin for 72 h. d) IL‐6 and TNF‐α mRNA levels in the kidneys were analyzed by qRT‐PCR. e) PAS staining (magnification: ×200, scale bar: 50 µm) of kidneys from control and MUTYH overexpression mice and tubular injury scores (right). f) TUNEL staining of kidneys of cisplatin‐treated control and MUTYH overexpression mice (scale bar: 20 µm; green: TUNEL, blue: DAPI). The quantitative analysis results are shown on the right. g) Images of the comet tail moment induced by cisplatin after type 1 or 2 human MUTYH overexpression in mice. h) Western blot analysis of KIM‐1, NGAL, and cleaved caspase 3 protein levels in the kidneys of cisplatin‐treated control and MUTYH overexpression mice. The results are shown as mean ± S.D (*n* = 6). ***P < 0.001, **P < 0.01, *P < 0.05 (one‐way ANOVA).

There are two types of human MUTYH: mitochondrial localization type 1 protein and nuclear localization type 2 protein.^[^ ^24^ ^]^ However, three types of mouse MUTYH (types a, b, and c) are generated by alternative splicing, and all three types of MUTYH are localized in both the nucleus and mitochondria.^[^ ^25^ ^]^ To understand the role of different types and localization of MUTYH in cisplatin‐induced AKI, human type 1 or 2 MUTYH plasmids were delivered to mice through tail vein high‐pressure injection, as previously described.^[^ ^26^ ^]^ Western blot and RT‐PCR showed that the expression of MUTYH was significantly increased in the kidneys 36 h after the injection of MUTYH plasmids (**Figure** **4**a,b). At 36 h after MUTYH plasmid injection, the mice were administered cisplatin for 72 h. Overexpression of type 2 human MUTYH, but not type 1 human MUTYH, markedly improved the renal function of cisplatin‐induced AKI mice compared to that of mice injected with vector plasmids, as evidenced by the lower Scr and BUN levels (Figure 4c), lower cisplatin‐induced renal inflammation (Figure 4d; and Figure S4a, Supporting Information), and lower tubular injury scores based on PAS staining (Figure 4e). Additionally, the reduced number of TUNEL‐positive cells (Figure 4f), comet tail moment (Figure 4g), and lower protein levels of KIM‐1, NGAL, and cleaved caspase‐3 (Figure 4h) showed that overexpression of human type 2 MUTYH plasmids in mice markedly attenuated the cisplatin‐induced tubular injury and DNA damage compared with that in mice injected with human type 1 MUTYH or control plasmids. To further analyze whether ectopic MUTYH overexpression could ameliorated cisplatin‐induced AKI in vivo, Flag‐tagged MUTYH1 and MUTYH2 plasmids were delivered to mice. Our results showed overexpression of Flag tagged type 2 MUTYH more than type 1 MUTYH ameliorated cisplatin‐induced AKI (Figure S4b–e, Supporting Information). These results showed that the overexpression of type 2, more than type 1 human MUTYH, ameliorated cisplatin‐induced AKI in vivo.

**Figure 5:**
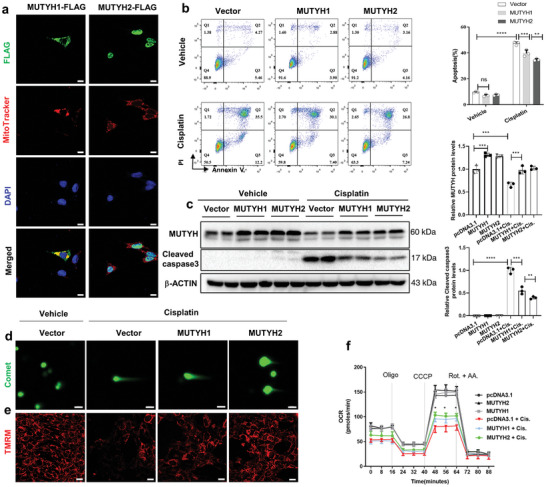
Overexpression of type 2 MUTYH ameliorates cisplatin‐induced apoptosis, DNA damage, and mitochondrial dysfunction in vitro. a) After transfection of mPTCs with type 1 or 2 human MUTYH, subcellular localization of human MUTYH were determined by immunofluorescence staining for anti‐FLAG. FLAG tag was added at the C‐terminus of the MUTYH protein. (Green: FLAG, red: Mitotracker, blue: DAPI; magnification: 630×, scale bar: 10  µm). b) Flow cytometry analysis of annexin V and PI staining and quantification of apoptosis in cisplatin‐treated (24 h) mPTCs after overexpression of type 1 or 2 human MUTYH. c) The protein levels of MUTYH and cleaved caspase3 in mPTCs overexpressing type 1 or 2 MUTYH induced by cisplatin were analyzed by Western blot. The graph on the right displays the results of the densitometry analysis. d) Representative images of the comet tail moment induced by cisplatin after type 1 or 2 human MUTYH overexpression in mPTCs. e) Fluorescent images of TMRM in MUTYH overexpressing mPTCs treated with cisplatin for 24 h. f) OCR in type 1 or type 2 MUTYH overexpression mPTCs measured using a Seahorse 96 XF‐analyzer. Cell experiments were performed three times, and data are expressed as mean ± S.D. (*n* = 3). ctrl: control, Cis: cisplatin; ***P < 0.001, **P < 0.01, *P < 0.05 (one‐ or two‐way ANOVA).

We aimed to elucidate whether overexpression of type 1 or 2 MUTYH has protective effects in mPTCs. mPTCs were transfected with FLAG‐tagged type 1 or 2 human MUTYH plasmids, and IF staining showed that type 1 human MUTYH was localized in the mitochondria and type 2 was localized in the nucleus (**Figure** **5**a). After plasmid transfection and overexpression for 36 h in mPTCs, the cells were treated with cisplatin for 24 h. The results showed that overexpression of type 1 and 2 MUTYH in mPTCs protected against cisplatin‐induced apoptosis, and overexpression of type 2 human MUTYH had a stronger protective effect than type 1, as evidenced by lower levels of apoptosis (Figure 5b,c), lower levels of 8‐oxoG accumulation both in nDNA and mtDNA (Figure S5a, Supporting Information), and comet tail moment (Figure 5e; and Figure S5b, Supporting Information) induced by cisplatin. Next, the protective effects of MUTYH overexpression on mitochondrial function were analyzed. The results showed that compared to type 1 MUTYH, type 2 overexpression better protected against cisplatin‐induced ROS production (Figure S5c, Supporting Information), MMP (Figure 5f; and Figure S5d, Supporting Information), and OCR downregulation (Figure 5g). These results showed that, compared to type 1, the overexpression of type 2 human MUTYH better protected against cisplatin‐induced DNA damage and mitochondrial dysfunction in mPTCs.

### 2.5 MUTYH Deficiency Aggravated Folic Acid‐Induced AKI, DNA Damage, and Mitochondrial Dysfunction

**Figure 6:**
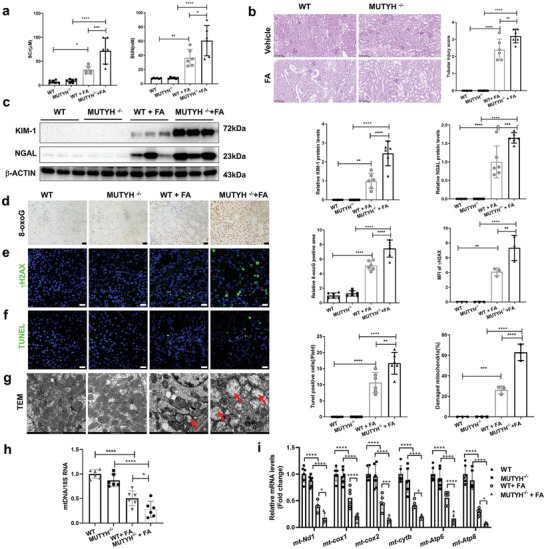
MUTYH deficiency aggravates folic acid‐induced AKI, DNA damage, and mitochondrial dysfunction. a) Scr and BUN levels in WT and MUTYH^−/−^ mice with or without 72 h FA treatment. b) PAS staining images (magnification: ×200) of kidneys from WT and MUTYH^−/−^ mice treated with FA and tubular injury scores (right). Five random fields were taken from each kidney. c) Western blot analysis of KIM‐1 and NGAL protein levels in the kidneys of FA‐induced WT and MUTYH^−/−^ mice. d) Immunohistochemical staining and quantification of 8‐oxoG levels (original magnification: 400×; scale bar: 20 µm). e) Immunofluorescent images and quantification of γH2AX levels in the kidneys of WT and MUTYH^−/−^ mice treated with FA for 72 h. (green: γH2AX, blue: DAPI; magnification: 400×, scale bar: 20 µm). f) TUNEL staining images and quantification in the kidneys of FA‐treated WT and MUTYH^−/‐^ mice (scale bar: 20 µm; green: TUNEL, blue: DAPI). g) Electron microscopy images and quantification of damaged mitochondria in renal tubular cells after 72 h FA treatment (scale bar: 1  µm, red arrow: damaged mitochondria). The quantitative analysis results are on the right. Mitochondria copy number h) and the genes encoded by the mitochondrial genome were analyzed by qRT‐PCR i). The results are shown as mean ± S.D. for six mice per group (*n* = 6). FA: folic acid; *P < 0.05, **P < 0.01, ***P < 0.001 (two‐way ANOVA).

To further explore the role of MUTYH in AKI, we investigated whether MUTYH deficiency aggravates FA‐induced AKI. As shown in **Figure** **6**a, the levels of Scr and BUN at 3 days after FA treatment were higher in MUTYH^−/−^ mice than in WT controls. PAS staining‐based tubular injury scores showed that MUTYH deficiency aggravated FA‐induced severe renal morphological injury, characterized by tubular cell dropout (Figure 6b). Consistently, the protein levels of NGAL and KIM‐1 were higher in the kidneys of MUTYH^−/−^ mice induced by FA treatment than WT mice after FA treatment for 72 h (Figure 6c). Furthermore, MUTYH deficiency aggravated FA‐induced DNA damage, as indicated by higher levels of 8‐oxoG both in nDNA and mtDNA (Figure 6d; and Figure S6, Supporting Information) and γH2AX (Figure 6e) in the kidneys of MUTYH^−/−^ mice, compared to levels in WT controls. Next, we examined renal tubular apoptosis in the kidneys of WT and MUTYH^−/−^ mice after FA treatment for 72 h. As shown in Figure 6f, TUNEL staining and statistical analysis revealed more apoptotic cells in the kidneys of MUTYH knockout mice after FA treatment than in WT controls. Electron microscopy images revealed that the number of damaged mitochondria with swollen and disrupted cristae in the renal tubular cells of FA‐treated MUTYH knockout mice was higher than that in WT controls (Figure 6g). Additionally, qRT‐PCR results showed that MUTYH deficiency aggravated the downregulation of mitochondrial copies and mRNA levels of mtDNA‐encoded genes compared with WT controls after FA treatment (Figure 6i). These results indicated that MUTYH deficiency aggravated FA‐induced nephrotoxicity, DNA damage, and mitochondrial dysfunction in mice.

### 2.6 E3 Ligase HUWE1 Mediated Ubiquitination Degradation of MUTYH

**Figure 7:**
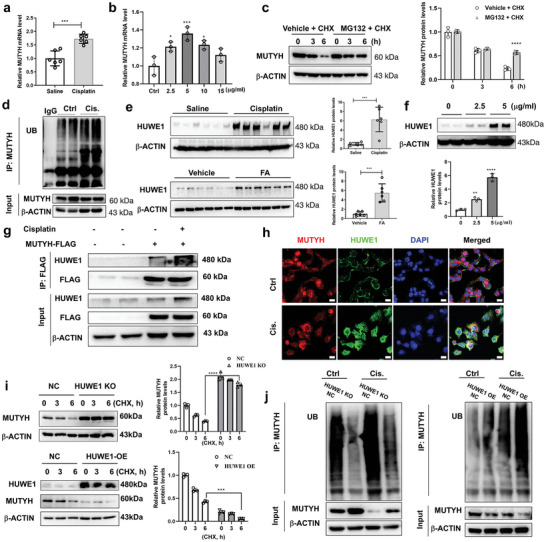
E3 ligase HUWE1 mediates ubiquitination degradation of MUTYH. a) qRT‐PCR analysis of MUTYH mRNA levels in the kidneys of cisplatin‐treated mice. b) qRT‐PCR analysis of MUTYH mRNA levels in mPTCs treated with cisplatin for 24 h. c) The protein stability of MUTYH in mPTCs treated with CHX and MG132 analyzed by Western blot. d) Ubiquitination levels of MUTYH in mPTCs analyzed using an ubiquitination assay. e) Western blot analysis of HUWE1 protein levels in the kidneys of cisplatin‐ or FA‐induced mice. f) Western blot and quantification of HUWE1 protein levels in mPTCs treated with cisplatin for 24 h. g) Interaction between HUWE1 and MUTYH analyzed using CO‐IP. h) Immunofluorescent co‐staining for MUTYH and HUWE1 in cisplatin‐treated mPTCs (red: MUTYH, green: HUWE1, blue: DAPI; magnification: 400×, scale bar: 20 µm). i) After different duration cycloheximide (CHX, 10 µg/ml) treatments, the protein stability of MUTYH in HUWE1 overexpression or knockout mPTCs was analyzed by Western blot. j) Ubiquitination levels of MUTYH in HUWE1 overexpression or knockout mPTCs treated with or without cisplatin (5 µg mL^−1^) for 24 h were analyzed using an ubiquitination assay. Cell experiments were performed three times, and data are expressed as mean ± S.D (*n* = 3 or 6). NC: negative control, KO: knockout, Cis: cisplatin; ***P < 0.001, **P < 0.01, *P < 0.05 (two‐way ANOVA).

Our results showed that the protein levels of MUTYH were downregulated in the kidneys of cisplatin‐ or FA‐induced AKI mice; however, its mRNA levels were increased in the kidneys of AKI mice and cisplatin‐treated mPTCs (**Figure** **7**a,b). Additionally, treatment with MG132, a proteasome inhibitor, significantly increased the stability of MTUYH in mPTCs (Figure 7c). Moreover, the ubiquitination level of MUTYH was analyzed using an in vivo ubiquitination assay, which showed that the levels of ubiquitin‐bound MUTYH increased in cisplatin‐treated mPTCs (Figure 7d). These results suggest that the downregulation of MUTYH protein levels may be due to posttranslational modifications, such as ubiquitination and degradation. To explore the possible mechanisms underlying the downregulation of MUTYH protein levels in the kidneys of AKI mice, we performed quantitative proteomic analysis to assess the differential ubiquitin and deubiquitinase protein expression in the kidneys of saline‐ and cisplatin‐treated mice. Compared to the saline group, cisplatin‐treated mice exhibited 19 differentially expressed ubiquitin or deubiquitin proteins (upregulated ≥1.5‐fold or downregulated ≤0.6‐fold) (Figure S7a, Supporting Information). Furthermore, Western blot showed that the protein levels of HUWE1, which is one of the ubiquitin E3 ligases of MUTYH^[^ ^22^ ^]^ were upregulated in the kidneys of cisplatin‐ or FA‐treated mice compared with that in controls (Figure 7e). Moreover, cisplatin upregulated the HUWE1 protein levels in mPTCs in a concentration‐dependent manner (Figure 7f) and cisplatin‐induced HUWE1 upregulation mainly distributed in the nucleus (Figure S7b, Supporting Information). A coimmunoprecipitation assay showed that FLAG‐tagged MUTYH could pull down HUWE1 in both cisplatin‐treated and untreated mPTCs (Figure 7g). Additionally, IF showed that HUWE1 costained with MUTYH in cisplatin‐treated mPTCs (Figure 7h). These results indicate that HUWE1 may bind to MUTYH. To confirm the role of HUWE1 in mediating the ubiquitination degradation of MUTYH, HUWE1 overexpression in mPTCs was constructed using a CRISPR/Cas9 SAM system. The cells were then treated with cycloheximide, a eukaryotic protein synthesis inhibitor, for different durations. Western blot showed that the protein stability of MUTYH was dramatically decreased when HUWE1 was overexpressed in mPTCs (Figure 7i). Additionally, HUWE1 knockout mPTCs were constructed using the CRISPR/Cas9 gene knockout method, and Western blot showed that the protein stability of MUTYH was markedly increased after HUWE1 knockout in mPTCs (Figure 7i). Moreover, the ubiquitination levels of MUTYH were analyzed, and the results showed that HUWE1 overexpression upregulated the levels of MUTYH ubiquitination, whereas MUTYH ubiquitination levels decreased after HUWE1 knockout in mPTCs (Figure 7j). These results suggest that HUWE1 mediates the ubiquitination and degradation of MUTYH and plays an important role in cisplatin‐ and FA‐induced AKI.

### 2.7 Inhibition of HUWE1 Ameliorated Cisplatin‐Induced AKI

**Figure 8:**
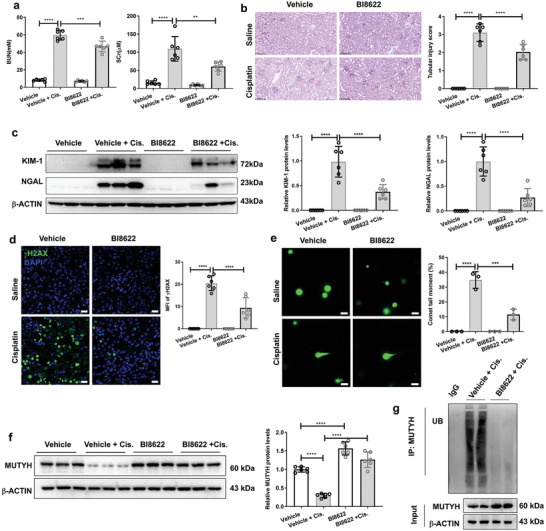
Inhibition of HUWE1 ameliorates cisplatin‐induced AKI. a) BUN and Scr levels in mice after i.p. injection of BI8622 and 72 h cisplatin treatment. b) PAS staining reveals that cisplatin‐induced renal tubular injury is greatly reversed by BI8622 (5 mg kg^−1^ d^−1^) treatment, and the renal tubular injury score was calculated (on the right) (magnification: 200×). c) Results of Western blot show that the protein levels of NGAL and KIM‐1 are significantly increased in the kidneys of cisplatin‐treated mice and are decreased after BI8622 treatment; the images were quantified using ImageJ (on the right). d) Immunofluorescent images and quantification of γH2AX levels in the kidneys of mice from different groups treated with cisplatin for 72 h. (green: γH2AX, blue: DAPI; magnification: 400×, scale bar: 20  µm). e) Images and quantification of the comet tail moment in the kidneys of mice from different groups treated with cisplatin for 72 h. (green: DNA; magnification: 200×, scale bar: 50  µm). f) The protein levels of MUTYH analyzed by Western blot. g) Ubiquitination levels of MUTYH in the kidneys of mice from different groups treated with cisplatin for 72 h were analyzed using an ubiquitination assay. Data are shown as mean ± S.D. for six or three mice per group (*n* = 3 or 6). Cis: cisplatin; ****P < 0.0001, ***P < 0.001, **P < 0.01 (two‐way ANOVA).

To examine whether the inhibition of HUWE1 could ameliorate cisplatin‐induced AKI, mice were treated with BI8622 (5 mg kg^−1^ d^−1^), an inhibitor of HUWE1, and a cisplatin‐induced AKI mouse model was constructed. As shown in **Figure** **8**a, BI8622 treatment significantly ameliorated cisplatin‐induced renal dysfunction. Histological analysis by PAS staining showed that administering BI8622 markedly ameliorated tubular injury compared to that in the vehicle group (Figure 8b). Additionally, Western blot showed that BI8622 treatment markedly decreased cisplatin‐induced NGAL and KIM‐1 protein levels (Figure 8c). IF showed that BI8622 treatment greatly ameliorated cisplatin‐induced DNA damage, as indicated by lower levels of γH2AX (Figure 8d) and comet tail moment (Figure 8e). BI8622 treatment upregulated the protein levels of MUTYH (Figure 8f) and decreased its ubiquitination level in the kidneys of cisplatin‐treated mice (Figure 8g). We also analyzed the BI8622 effects in MUTYH KO mice. Our results indicated that the MUTYH knockout significantly diminished the protective effects of BI8622 in mice (Figure S8, Supporting Information). Nonetheless, BI8622 exhibited a mild protective effect in MUTYH KO mice, as evidenced by BUN levels (Figure S8a, Supporting Information), tubular injury scores derived from PAS staining (Figure S8b, Supporting Information), and KIM‐1 protein levels (Figure S8c, Supporting Information). The roles of HUWE1 in cisplatin‐induced AKI were confirmed in HUWE1 knockout mPTCs. Our results showed that HUWE1 knockout markedly ameliorated cisplatin‐induced apoptosis in mPTCs (Figure S9a, Supporting Information). The EC50 of BI8622 on mPTCs was 17.35 µm (Figure S9b, Supporting Information). Moreover, the protective effects of BI8622 were markedly attenuated by HUWE1 knockout in mPTCs (Figure S9c, Supporting Information). These results suggest that the inhibition or knockout of HUWE1 ameliorates cisplatin‐induced AKI.

## 3 Discussion

DNA damage includes DNA‐strand break (single‐or double), base losses, and mismatched base pairs. Previous studies have demonstrated that ATM and ATR, two key DDR proteins, protect against progressive kidney diseases, including AKI.^[^ ^6^, ^27^ ^]^ However, no study has reported the role of MUTYH in AKI. In this study, we provide new insights into the role of MUTYH in the pathogenesis of AKI using MUTYH knockout mice, overexpression of different types of human MUTYH in vivo, and cultured tubular cells with MUTYH knockout or overexpression in vitro, suggesting that MUTYH plays a protective role in cisplatin‐ or FA‐induced AKI by preventing cellular DNA damage and mitochondrial dysfunction.

The DNA glycosylase MUTYH plays an important role in genetic integrity maintenance, the incorrect repair of A: 8‐oxoG mispairs in MUTYH^−/−^ mice shows an increase in mutation frequency with a higher incidence of tumor formation when the mice are more than 18 months old.^[^ ^15b^ ^]^ In this study, 6–8 weeks old mice were used to construct AKI models. Additionally, Renal tubular cells are the primarily cells affected by AKI insults.^[^ ^28^ ^]^ Our results also showed MUTYH was mainly expressed in renal tubule cells and decreased significantly after cisplatin treatment in kidneys of mice. Almost all types of tubular insults can induce DNA damage in tubular cells through alkylation, oxidative stress, or ROS release.^[^ ^6d^ ^]^ 8‐oxoG is one of the most prevalent ROS‐induced oxidative DNA lesions^[^ ^6^, ^29^ ^]^ At baseline, we found that the kidney functions of MUTYH^−/−^ mice are normal even through the levels of 8‐oxoG are increased compared with those of WT mice. Our findings indicate that DNA oxidation constantly occurs in AKI as the levels of 8‐oxoG increase in the kidneys of cisplatin‐induced AKI mice. Previous studies have shown that the BER signaling coordinated by the DNA glycosylase MUTYH and OGG1 under tight control can repair damaged DNA.^[^ ^11b^ ^]^ In this study, our results showed that only deficiency of the BER protein, MUTYH, aggravated cisplatin‐induced DNA damage and AKI, as evidenced by increased 8‐oxoG, γH2AX foci, markers of kidney injury, and cell death, when compared with WT mice. As a BER protein, MUTYH deficiency also enhanced the generation of DSBs perhaps through overproduction of ROS. Additionally, a recent study found an upregulation of DSBs generation due to the accumulation of 8‐oxoG.^[^ ^30^ ^]^ These results suggest that the BER machinery plays a protective role in AKI and that its deficiency may lead to the pathological progression of AKI.

Eukaryotic cells have two genomes, nuclear and mitochondrial, both of which are vulnerable to damage under oxidative stress.^[^ ^24^, ^25^ ^]^ Therefore, cells must develop either two distinct repair enzymes or transport the same enzymes into separate organelles. MUTYH is an enzyme encoded by nuclear genes but is localized in both the nucleus and mitochondria and plays an important role in the repair of oxidative DNA damage.^[^ ^24^, ^25^ ^]^ Few studies have reported the differential subcellular localization of human MUTYH.^[^ ^24^, ^31^ ^]^ Renal tubule cells are rich in mitochondria, and mitochondrial dysfunction characterized by reduced MMP, OCR, and the expression of components of the electron transport chain is the major pathological factor of AKI; thus, studies have focused on improving mitochondrial function to treat AKI.^[^ ^32^ ^]^ The mitochondrial localization type 1 protein of MUTYH was anticipated to play a more important role than the nuclear localization type 2 protein in protecting against cisplatin‐induced AKI. However, our results showed that overexpression of type 2 MUTYH, but not type 1, in vivo ameliorated cisplatin‐induced AKI, although overexpression of both type 1 and type 2 MUTYH protected against cisplatin‐induced apoptosis and mitochondrial dysfunction in vitro. Additionally, type 2 human MUTYH was better than type 1 at protecting against cisplatin‐induced mitochondrial dysfunction in mPTCs. These results suggest that the stability of the nuclear genome is more important than that of the mitochondrial genome in protecting against cisplatin‐induced AKI. The protein composition of the mitochondria may explain this phenomenon. More than 1000 proteins comprise the mitochondrial protein network, and the mitochondrial genome encodes only 13 proteins, which are components of the respiratory chain or ATP synthase, while the other mitochondrial proteins are encoded by nuclear genes and transported into the mitochondria.^[^ ^33^ ^]^ The expression of nuclear‐encoded mitochondrial proteins is affected by the nuclear genome stability.^[^ ^34^ ^]^ The stability of the nuclear genome may play a more important role in maintaining mitochondrial function than that of the mitochondrial genome. The results of our study suggest a focus on improving the stability of the nuclear genome instead of that the mitochondrial genome, providing a new strategy for the treatment of cisplatin‐induced AKI.

Previous studies have revealed that cisplatin and its metabolites can easily cross‐link nuclear and mitochondrial genomic DNA, induce DNA damage, and inhibit DNA synthesis in kidney tubular cells.^[^ ^35^ ^]^ As expected, MUTYH, a BER protein, plays a role in cisplatin‐induced nephrotoxicity. In this study, we extended the study to an FA‐induced AKI model in addition to the cisplatin‐induced AKI model. FA‐induced AKI has been described in humans^[^ ^36^ ^]^ and an animal experimental model recapitulates all major processes in human AKI, such as renal cell death and inflammation.^[^ ^37^ ^]^ Our results showed that MUTYH deficiency aggravated FA‐induced DNA damage, mitochondrial dysfunction, cell death, and AKI in mice, which was consistent with findings in a cisplatin‐induced animal model. These results suggest that the BER machinery also plays a protective role in FA‐induced AKI and that MUTYH may play a protective role in various types of AKI.

Differential protein expression in the kidneys of cisplatin‐treated AKI mice was analyzed using proteomics, and the results showed that the protein level of HUWE1 increased in AKI mice. A previous study found that HUWE1 is an ubiquitin E3 ligase of MUTYH that mediates the ubiquitination and degradation of MUTYH in tumor cells.^[^ ^22^ ^]^ Our results showed that protein levels of the E3 ligase HUWE1 were upregulated in the kidneys of cisplatin‐ or FA‐treated mice, and overexpression of HUWE1 aggravated cisplatin‐induced tubular cell death. Knockdown of HUWE1, also known as ARF‐binding protein 1/ARF‐BP1 or MULE, leads to DNA polymerase beta (Pol β) accumulation and increased DNA repair.^[^ ^38^ ^]^ In this study, we found that the protein stability of MUTYH was also regulated by HUWE1 in renal tubular cells. Furthermore, treatment with the HUWE1 inhibitor BI8622 protected against cisplatin‐induced AKI in mice. These results suggest that HUWE1 may serve as a therapeutic target for AKI, although the detailed underlying mechanisms require further investigation.

Our study had some limitations. For example, the role of MUTYH in clinical settings remains to be explored further, because only four renal samples from human AKI patients were collected and used to analyze MUTYH expression levels in this study. And these four samples only involve children with AKI, the expression of MUTYH in adult patients with AKI needs to be examined further. In this study, we only analyzed the protein levels of MUTYH in kidney samples from patients with AKI using IHC staining. Future research exploring the role of MUTYH in human kidney injury could be done using human kidney organoid. In addition, the role and detailed mechanisms of the ubiquitin E3 ligase HUWE1 in the pathological progression of AKI require further investigation. Our results indicated that the MUTYH knockout largely diminished the protective effects of BI8622 in mice. However, BI8622 treatment still exhibited a mild protective effect in MUTYH KO mice, which suggests the involvement of additional drug targets beyond MUTYH in this experimental setting. Thus, further investigation is warranted. Moreover, a previous study reported that polyubiquitylation of Pol β by HUWE1 is modulated by ARF,^[^ ^38^ ^]^ and that ARF or other regulatory factors in HUWE1 that modulate the cellular levels of MUTYH require further investigation.

## 4 Conclusions

In summary, this study demonstrates that MUTYH, a DNA glycosylase, plays an important role in the repair of oxidative DNA damage and is downregulated in the renal tubular epithelium of AKI patients and cisplatin‐or folic acid‐induced AKI mice. We showed that upregulation of the ubiquitin E3 ligase HUWE1‐mediated ubiquitination degradation of MUTYH markedly aggravated AKI, possibly by aggravating DNA damage and mitochondrial dysfunction in vivo and in vitro. Additionally, our findings show that overexpression of type 2 human MUTYH is better than that of type 1 MUTYH in protecting against cisplatin‐induced mitochondrial dysfunction. The development of effective and safe agonists of MUTYH or inhibitors of HUWE1 and clinical trials of these agents in patients with AKI are attractive for AKI therapy. The findings from our research not only help to understand the pathogenesis of AKI, but also provide possible new therapeutic strategies for the treatment of AKI.

## 5 Experimental Section

### AKI Patients

Four patients with AKI, verified by renal biopsy, at the Children's Hospital of Nanjing Medical University were enrolled in this study. Clinical parameters, including age, sex, cause of AKI, and serum creatinine (Scr) levels at the peak and time of renal biopsy, were collected and are listed in Table S1 (Supporting Information). AKI was diagnosed based on the KDIGO (kidney disease: Improving Global Outcomes) criteria. Paracarcinoma kidney samples from four patients who underwent renal carcinoma resection were collected (Table S2, Supporting Information) and used as controls for the MUTYH assay, after the renal samples were verified to have no evidence of kidney injury through routine pathological examination. The Committee on Research Ethics of Nanjing Medical University approved the protocol for the use of patient samples in this study (registration number: 201801158‐2). Informed consent was obtained from all participants.

### MUTYH^−/−^ Mice and Animal Models of AKI

MUTYH^+/−^ mice with a C57BL/6J genetic background were previously established (provided by Prof. Yusaku Nakabeppu).^[^ ^15b^ ^]^ MUTYH^−/−^ mice and WT littermates were bred and genotyped, as previously described,^[^ ^15b^ ^]^ at the Laboratory Animal Center of Nanjing Medical University (Nanjing, China). Mice were maintained in a standard specific pathogen‐free (SPF) animal room with 12 h light/dark cycles and free access to food and water. To confirm the role of the MUTYH gene in cisplatin‐induced AKI, four groups (*n* = 6 per group) of mice were established: WT mice (WT group), MUTYH^−/−^ mice (MUTYH^−/−^ group), cisplatin‐treated WT mice (WT + cisplatin group), and cisplatin‐treated MUTYH^−/−^ mice (MUTYH^−/−^ + cisplatin group). Mice in the cisplatin group received a single intraperitoneal injection of 20 mg kg^−1^ cisplatin (QILU Pharmaceutical, China, H20023461) in saline, while the control group received an equal amount of saline. To further validate the overexpression of different types of human MUTYH in cisplatin‐induced AKI, 60 µg of type 1 human MUTYH plasmids (pcDNA3.1‐MUTYH1), type 2 human MUTYH plasmids (pcDNA3.1‐MUTYH2), or vector control plasmids (pcDNA3.1) were dissolved in 2 mL of saline and administered to mice within 10 s though tail vein injection, as previously described.^[^ ^26^ ^]^ After 36 h, the mice (*n* = 6 per group) were administered cisplatin, as described above. The effect of BI8622 (HY‐120929; MedChemExpress) on cisplatin‐induced AKI was also examined. Mice were assigned to four groups: vehicle group (5% DMSO + 40% PEG300 + 5% Tween 80 + 50% ddH_2_O; *n* = 6), BI8622 treatment group (BI8622, *n* = 6), cisplatin‐treated group (vehicle + cisplatin, *n* = 6), and cisplatin plus BI8622 group (BI8622 + cisplatin, *n* = 6). Mice in the BI8622 treatment group received intraperitoneal injections of BI8622 (5 mg kg^−1^ d^−1^) 6 h before cisplatin treatment and then once daily until the mice were euthanized. To test the effects of BI8622 on MUTYH knockout mice, mice were assigned to six groups (*n* = 6) as indicated in figure legend. In the FA‐induced AKI model, mice were assigned to four groups (*n* = 6 per group): WT mice (WT group), MUTYH^−/−^ mice (MUTYH^−/−^ group), FA‐treated WT mice (WT+FA group), and FA‐treated MUTYH^−/−^ mice (MUTYH^−/−^+FA group). The mice received a single intraperitoneal injection of 240 mg kg^−1^ FA (Sigma, St. Louis, MO) in 0.15 m NaHCO_3_ or vehicle. The mice in all groups were euthanized after receiving cisplatin or FA treatment for 72 h. Serum and kidneys were collected and stored at −80 °C for further analysis. For histological analysis, kidney tissues were fixed in 4% paraformaldehyde. Scr and blood urea nitrogen (BUN) levels were analyzed using an automatic biochemical analyzer at the Children's Hospital of Nanjing Medical University. The mice used in this study were 6–8 weeks old male mice. All animal procedures were approved by the Institutional Animal Care and Use Committee of Nanjing Medical University (registration number: IACUC 14030112‐2) according to ARRIVE guidelines.^[^ ^39^ ^]^

### Cell Culture and Treatment

Mouse renal proximal tubular cells (mPTCs; CRL‐3361, ATCC, USA) were cultured in DMEM/F‐12 medium (Gibco, 319‐075‐CL) supplemented with 10% fetal bovine serum (FBS; GIBCO, 26170035), penicillin (100 U mL^−1^), and streptomycin (100 µg mL^−1^) and maintained at 37 °C in 5% CO_2_ in a humidified incubator. CRISPR/Cas9 was used to knockout MUTYH or HUWE1 in mPTCs; sgRNAs targeting MUTYH or HUWE1 were cloned into pSpCas9(BB)‐2A‐Puro (PX459) v2.0, which was a gift from Feng Zhang (Addgene plasmid #62988), as previously described,^[^ ^40^ ^]^ and the sequences are listed in Table S3 (Supporting Information). The sequenced CRISPR/Cas9 plasmids targeting MUTYH or HUWE1 were transfected into mPTCs with PolyJet DNA transfection reagent (SignaGen, SL100688), and positive cells were selected using puromycin (2 µg mL^−1^) for 3 days prior to clonal expansion. PX459 was used as a negative control. For MUTYH overexpression in mPTCs, different types of FLAG‐tagged human MUTYH plasmids were transfected into mPTCs with PolyJet DNA transfection reagent (SignaGen, SL100688) and overexpressed for 36 h. As the protein molecular weight of HUWE1 is >480 kDa, the CRISPR/Cas9 Synergistic Activation Mediator (SAM) system was used to activate the expression of HUWE1 in mPTCs, as previously described.^[^ ^41^ ^]^ The sequences of the guide RNA are listed in Table S3 (Supporting Information). mPTCs were treated with cisplatin (5 µg mL^−1^) for 24 h or for the indicated times.

### Isolation of Primary Renal Epithelial Cells

Primary renal epithelial cells were isolated from wild‐type (WT) or MUTYH knockout (KO) mice based on the previous study.^[^ ^26^ ^]^ In brief, male mice aged 4–6 weeks were euthanized, and their kidneys were promptly harvested and immersed in cold Hank's Balanced Salt Solution (HBSS) supplemented with 1% penicillin and streptomycin. Subsequently, the kidneys were minced into ≈1 mm^3^ pieces and digested in 5 mL of HBSS containing collagenase I (2 mg mL^−1^) for 30 min at 37 °C. The resulting solution was filtered through a 100 µm nylon mesh, followed by centrifugation at 900 g for 10 min at 4 °C. The primary cells were cultured in RPMI‐1640 medium supplemented with 10% fetal bovine serum (FBS), 20 ng mL^−1^ epidermal growth factor (EGF, Peprotech, 315‐09), and 100 units mL^−1^ penicillin and 100 µg mL^−1^ streptomycin, maintained at 37 °C with 5% CO_2_ in a humidified incubator. The cells were utilized after 7 days of cultivation.

### qRT‐PCR

Total RNA was extracted using TRIzol reagent (TAKARA, Dalian, China; 9108). CDNA was reverse transcribed from 1 µg of total RNA using a reverse transcriptase M‐MLV kit (TAKARA, 2641A). Primers (Table S3, Supporting Information) were designed and synthesized by Tsingke (Nanjing, China). QRT‐PCR was performed using the SYBR Green master mix (Vazyme, Nanjing, China; q111‐02/03) and the QuantStudio 3 Real‐Time PCR System (Applied Biosystems, Foster City, CA). The relative levels of messenger RNA (mRNA) expression were corrected by β‐actin, then converted to fold changes using the 2^−ΔΔCt^ method, as previously described.^[^ ^42^ ^]^

### mtDNA Copy Number

Total DNA was extracted from kidney tissues using a DNeasy Tissue Kit (69506; QIAGEN Sciences, Germantown, MD) according to the manufacturer's instructions. QRT‐PCR was performed to detect the mtDNA copy number; the primers used were the same as those used in a previous study^[^ ^43^ ^]^ and are listed in Table S3 (Supporting Information). Relative mtDNA copy numbers were normalized to the 18S rRNA gene and converted to fold changes using the 2^−ΔΔCt^ method, as previously described.^[^ ^43^ ^]^

### Western Blot

Samples of kidney tissue or cells were homogenized in protein lysis buffer (50 mm Tris‐HCl, 250 mm NaCl, 0.5% Triton X‐100, 50 mm NaF, 2 mm EDTA, and 1 mm Na_3_VO_4_) supplemented with a 1× protease inhibitor cocktail (Roche, 04693132001) for 30 min on ice at 4 °C and then centrifuged at 12 000 rpm for 15 min. The Bradford method was used to measure the protein concentration, and 30 µg of total protein from each sample was analyzed by standard Western blot. The primary antibodies against NGAL (Abcam; ab63929, 1:1000), KIM‐1 (R&D systems, AF1817, 1:1000), cleaved caspase‐3 (Cell Signaling Technology; 9664, 1:1000), BAX (Proteintech; 50599‐2‐Ig, 1:1000), ATPB (Proteintech; 17247‐1‐AP, 1:1000), SOD2 (Proteintech; 24127‐1‐AP, 1:1000), MUTYH (Abcam; ab228722, 1:1000), HUWE1(Cell Signaling Technology; 5695S, 1:1000), Flag (Sigma; 19374‐1‐AP, 1:1000), and β‐actin (Proteintech; 60008‐1‐Ig, 1:1000) were diluted in 3% nonfat milk prepared in tris‐buffered saline (TBST, 0.1% Tween 20). Peroxidase‐conjugated goat antirabbit (Beyotime; A0208), antimouse (Beyotime; A0216), or antigoat (Beyotime; A0181) secondary antibodies were used at a 1:1000 dilution. Immunoblotted images were detected by an enhanced chemiluminescence detection system (Bio‐Rad, Hercules, CA). Densitometric analysis of the immunoblotted bands was performed using ImageJ software (National Institutes of Health, USA).

### Ubiquitylation Assays

To examine the ubiquitination of overexpressed MUTYH, mPTCs overexpressing HUWEI were constructed using the CRISPR/Cas9 SAM system, as described above. Then, mouse MUTYH‐FLAG and HA‐ubiquitin plasmids were transfected into HUWE1 overexpression or NC mPTCs using PolyJet DNA transfection reagent (SignaGen, SL100688). After 24 h, the cells were treated with 10 µm MG132 for 6 h before harvest. MUTYH was immunoprecipitated with anti‐FLAG (Sigma, F1804) and detected by Western blot using an antibody against ubiquitin (Ub; Cell Signaling Technology, 3936, 1:1000). To examine the ubiquitination levels of MUTYH in the kidneys, kidney tissues from cisplatin‐ or saline‐treated mice were lysed with NP40 lysis buffer, and MUTYH was immunoprecipitated with anti‐MUTYH (Abcam; ab228722) and detected by Western blot with an antibody against Ub.

### Histological Analysis of Kidneys

PAS staining was performed on 4‐µm kidney sections. Renal histology was analyzed using PAS staining, and histological changes in the kidneys were evaluated by calculating the percentage of damaged renal tubules that displayed cell lysis, brush border loss, or cast formation. Tissue pathological damage to the kidneys was scored from 0 to 4: 0, no abnormalities; 1, <25% renal tubules damaged; 2, 25%–50% renal tubules damaged; 3, 50%–75% renal tubules damaged; and 4, >75% renal tubules damaged.^[^ ^44^ ^]^ Images of each sample were captured using an Olympus BX51 microscope (Olympus, Center Valley, PA), and five random visual fields of each sample were quantified.

### IHC and Immunofluorescent Staining (IF)

After deparaffinization and rehydration, 4 µm paraffin‐embedded kidney sections were boiled in 500 mL 1× improved Citrate Antigen Retrieval Solution (Beyotime, P0083) for 1 min and cooled to room temperature (RT). The sections were blocked with 3% H_2_O_2_ for 10 min, washed three times with TBST buffer, incubated with 10% normal goat serum for 60 min at RT, and incubated with primary rabbit antibody against MUTYH (Abcam; ab228722, 1:100), F4/80 (CST; 70076, 1:150), or 8‐oxoG (Abcam; ab48508, 1:100) overnight at 4 °C. After washing three times with TBST buffer, kidney sections were incubated with horseradish peroxidase‐conjugated secondary antibodies for 60 min at RT. Finally, a DAB kit (ZLI‐9018, Zsbio, China) was used to stain the localization of peroxidase conjugates, and images were captured using an Olympus BX51 microscope (Olympus, Center Valley, PA). The positive areas of the IHC images were analyzed using the ImageJ software (National Institutes of Health, USA). IF was performed to detect the phosphorylation of the histone H2A variant H2AX (γH2AX) in the kidneys. Briefly, the antigen retrieval used for the kidney sections was the same as that for IHC staining. Kidney sections were then incubated with the primary rabbit antibody against γH2AX (Cell Signaling Technology, 9718S, 1:400) overnight at 4 °C. An Alexa Fluor 488‐conjugated goat antirabbit IgG (H+L) cross‐adsorbed secondary antibody (Thermo Fisher Scientific, A‐11008, 1:500) was used, according to the manufacturer's instructions. The nuclei were counterstained with DAPI (Beyotime, P0131). Images were obtained using a LSM710 confocal microscope (Carl Zeiss, Germany). For IF, mPTCs were fixed with 4% paraformaldehyde for 10 min, permeabilized with 0.1% Triton X‐100 for 10 min, blocked with 1% BSA for 1 h, and labeled with primary rabbit antibody against γH2AX (Cell Signaling Technology, 9718S, 1:400) or FLAG (Cell Signaling Technology, 14793S, 1:100) overnight at 4 °C; the remaining steps were the same as above. Quantification was performed using ImageJ software (National Institutes of Health, USA).

### Terminal Deoxyribonucleotidyl Transferase‐Mediated dUTP‐Digoxigenin Nick End Labeling (TUNEL) Assay

In situ cell death in the kidney was analyzed using a TUNEL BrightGreen Apoptosis Detection Kit, according to the manufacturer's instructions (A112‐01/02/03, Vazyme, China). After staining, images were captured using a laser scanning confocal microscope (Carl Zeiss LSM710, Germany). Five random visual fields of each sample were examined, and the number of apoptotic cells (i.e., TUNEL‐positive cells) was counted.

### Conventional Transmission Electron Microscopy

To evaluate mitochondrial morphology, renal cortex tissues were fixed in 1.25% glutaraldehyde/0.1 m phosphate buffer and postfixed in 1% OsO_4_/0.1 m phosphate buffer. Using a microtome, ultrathin (60 nm) sections of the renal cortex were cut, placed on copper grids, stained with uranyl acetate and lead citrate, and examined and photographed using an electron microscope (JEOL JEM‐1010; Tokyo, Japan). Five random visual fields of each sample were examined, and the percentage of damaged mitochondria (characterized by swollen and disrupted cristae) was quantified, as previously described.^[^ ^45^ ^]^

### Immunodetection of 8‐oxoG

The levels of 8‐oxoG were also analyzed using IF staining. Paraffin‐embedded kidney sections (3 µm) were deparaffinized and rehydrated. The DNA was denatured by boiling in a 10 mm citric acid buffer in a microwave for 15 min. Subsequently, the slides were treated with RNase A (5 mg mL^−1^) at 37 °C for 1 h, in accordance with a previous study.^[^ ^46^ ^]^ Then the slides were stained with a mouse anti‐8‐oxo‐dG primary antibody (N45.1, MOG020P, JaICa) and a proper Alexa Fluor 488‐labeled secondary antibody as previous study described.^[^ ^47^ ^]^ To analyze the levels of 8‐oxoG in nuclear DNA (nDNA) and mitochondrial DNA (mtDNA) both in vivo and in vitro, immunodetection of 8‐oxoG was performed following the protocols established in previous studies.^[^ ^47^, ^48^ ^]^ To analyze the levels of 8‐oxoG in mtDNA, frozen kidney sections (5 µm) were initially treated with RNase A (5 mg mL^−1^) and subsequently subjected directly to IF staining using an anti‐8‐oxo‐dG antibody (ab62623, abcam). In contrast, for the analysis of 8‐oxoG in nDNA, the frozen kidney sections (5 µm) underwent RNase treatment followed by a nDNA denaturation step with 2 m HCl prior to IF staining with the anti‐8‐oxo‐dG antibody (ab62623, abcam). Additionally, the levels of 8‐oxoG in nDNA and mtDNA in mPTCs were also analyzed separately. Briefly, the cell slides were prepared and underwent RNase treatment followed by a DNA denaturation step (50 mm NaOH for mtDNA for 7 min, 2 m HCl for nDNA for 10 min) prior to stain with 8‐oxoG with the anti‐8‐oxo‐dG primary antibody (ab62623, abcam) and a 488‐labeled secondary antibody. Images were obtained using a LSM710 confocal microscope.

### Apoptosis Assay

Cisplatin‐induced apoptosis of mPTCs was analyzed by double staining with propidium iodide (PI) and annexin V‐FITC using an apoptosis detection kit (BD Biosciences, 556547, San Diego, CA). Briefly, after treatment with cisplatin for 24 h, mPTCs were trypsinized with EDTA‐free trypsin, harvested, washed with cold PBS, and labeled with PI and fluorescein isothiocyanate (FITC), according to the manufacturer's protocol. Finally, apoptosis was measured using a flow cytometer (BD Biosciences, San Diego), and the results were analyzed using FlowJo software (TreeStar, Ashland, OR).

### Detection of Mitochondrial Membrane Potential and Mitochondrial ROS

The mitochondrial membrane potential (MMP) of mPTCs was analyzed by staining with tetramethylrhodamine methyl ester (TMRM, Thermo Fisher, I34361), a cell‐permeant dye that accumulates in active mitochondria with intact membrane potentials, according to the manufacturer's instructions. Briefly, mPTCs were seeded on sterile poly‐L‐lysine‐coated glass flakes, grown to 70% density, and treated with cisplatin for 24 h. After treatment, the cells were incubated with TMRM in the dark for 30 min at 37 °C and washed three times with PBS. Then, images were captured by laser scanning confocal microscopy (Carl Zeiss, LSM710, Germany), and the mean fluorescence intensity (MFI) of TMRM was analyzed and quantified using Image J software. To measure the mitochondrial ROS production, mPTCs were stained with MitoSOX red mitochondrial superoxide indicator (Thermofisher, M36008) after treatment with cisplatin for 12 h, according to the manufacturer's instructions. Fluorescent images were captured using a confocal microscope (Carl Zeiss LSM710, Germany), and the MFI of MitoSOX was analyzed using a flow cytometer (BD Biosciences, San Diego, CA). To measure the total ROS production, mPTCs were analyzed with a Reactive Oxygen Species Assay Kit (beyotime, S0033S) after treatment with cisplatin for 12 h, according to the manufacturer's instructions, the MFI of ROS was analyzed using a flow cytometer.

### Measurement of OCR Using an XF‐96 Flux Analyzer

The OCR of the mPTCs was analyzed using a Seahorse XF‐96 Extracellular Flux Analyzer (Seahorse Bioscience, Copenhagen, Denmark). Briefly, mPTCs with MUTYH knockout or overexpression were treated with cisplatin. Then, the cells were incubated at 37 °C in an incubator without CO_2_ for 1 h before measuring the basal OCR. The basal oxygen consumption was recorded for 16 min, and the OCR was measured following successive additions of the following mitochondrial inhibitors: oligomycin (oligo, 1 µm), which blocks mitochondrial complex V, was used to analyze the ATP‐synthesis coupling efficiency; the uncoupler carbonyl cyanide 4‐trifluoromethoxy‐phenylhydrazone (CCCP, 0.5 mm) was used to analyze the spare respiratory capacity; and a mixture of rotenone (Rot, 0.5 µm) and antimycin A (AA, 0.5 µm), which are complex I and complex III inhibitors, respectively, was used to assess the nonmitochondrial oxygen consumption.

### Comet Assays

Alkaline comet assays were performed to measure the single‐strand breaks (SSBs) and double‐strand breaks (DSBs) in DNA, as these DNA breaks increase DNA migration out of the nucleus during electrophoresis. Comet assays were performed using comet assay kits (Abcam, ab238544), according to the manufacturer's protocol. Images were obtained using a LSM710 confocal microscope (Carl Zeiss, Germany). The tail moment, including the amount of DNA that left the nucleus and the migration distance, reflected DNA strand breakage and was quantified using the CASP Comet Assay Software 1.2.3.

### Proteomic Analysis

Proteomic analyses were performed using PTM Biolabs (PTM Biolabs, China). Briefly, kidney protein samples from cisplatin‐ or saline‐treated mice were prepared and diluted for trypsin digestion. The peptides were loaded onto a nitrogen solubility index (NSI) source followed by tandem mass spectrometry (MS/MS) using Q Exactive (Thermo Fisher Scientific, San Jose, CA) coupled online to an ultraperformance liquid chromatography (UPLC) system. Label‐free quantification of MS/MS data was carried out using MaxQuant software (version v.1.6.6.0), as described previously,^[^ ^49^ ^]^ with the FDR (False Discovery Rate) adjusted to <1%, and the minimum score for modified peptides set to >40.

### Statistical Analysis

All results are presented as the mean ± standard deviation (SD). Statistical significance between groups was determined by the unpaired Student's *t*‐test, one or two‐way ANOVA using GraphPad (6.01, San Diego, CA). Significance was set at *P*‐values <0.05.

### Ethical Statement

All animal procedures were approved by the Institutional Animal Care and Use Committee of Nanjing Medical University (registration number: IACUC 14030112‐2) according to ARRIVE guidelines. The Committee on Research Ethics of Nanjing Medical University approved the protocol for the use of patient samples in this study (registration number: 201801158‐2). Informed consent was obtained from all participants.

## Conflict of Interest

The authors declare no conflict of interest.

## Author Contributions

Y.Y.: Conceptualization; Data curation; Formal analysis; Funding acquisition; Investigation; Methodology; Project administration; Writing – original draft. P.W.: Formal analysis; Methodology; Project administration. K.Z.: Project administration; Data curation; Formal analysis; Investigation. W.Z.: Project administration; Supervision; Validation; Visualization. S.L. and J.O.: Supervision; Validation; Visualization. M.B.: Resources; Software and G.D.: Supervision; Validation. S.H., Z.J., and A.Z.: Conceptualization; Project administration; Writing – review & editing; funding acquisition. All authors read and approved the final manuscript.

## Supporting information



Supporting Information

## Data Availability

The data that support the findings of this study are available from the corresponding author upon reasonable request.
