# Supplementary material for: HUWE1‐Mediated Degradation of MUTYH Facilitates DNA Damage and Mitochondrial Dysfunction to Promote Acute Kidney Injury
Source: Adv Sci (Weinh). 2025 Feb 8;12(13):2412250. doi: 10.1002/advs.202412250 (PMC11967787; doi:10.1002/advs.202412250)
Supplement: Supplementary file 1 — Supporting Information [file ADVS-12-2412250-s001.docx]

**Supplementary Information for**

**HUWE1-Mediated Degradation of MUTYH Facilitates DNA Damage and Mitochondrial Dysfunction to Promote Acute Kidney Injury**

Yunwen Yang, Peipei Wang, Kaiqian Zhou, Wen Zhang, Suwen Liu, Jing Ouyang, Mi Bai, Guixia Ding*, Songming Huang*, Zhanjun Jia*, and Aihua Zhang*

**Figure S1.** The experission of MUTYH in patients and mice kidneys.

**Figure S2.** MUTYH knockout aggravates cisplatin-induced AKI.

**Figure S3.** QRT-PCR were performed to confirm the successful construction of MUTYH^-/-^ mPTCs and the quantification results of Figure 3.

**Figure S4.** Overexpression of Flag tagged type 2 MUTYH ameliorated cisplatin-induced AKI.

**Figure S5.** Immunofluorescent images of 8-oxoG and the quantification results of Figure 5.

**Figure S6.** Immunofluorescent images of 8-oxoG in FA mice.

**Figure S7.** The expression of HUWE1 in vivo and in vitro.

**Figure S8.** The effects of BI8622 on MUTYH KO mice.

**Figure S9.** Inhibition or knockout of HUWE1 ameliorated cisplatin-induced injury in mPTCs.

**Table S1.** Clinical data of AKI patients.

**Table S2.** Clinical data of renal tumor patients

**Table S3.** Primer Sequences.


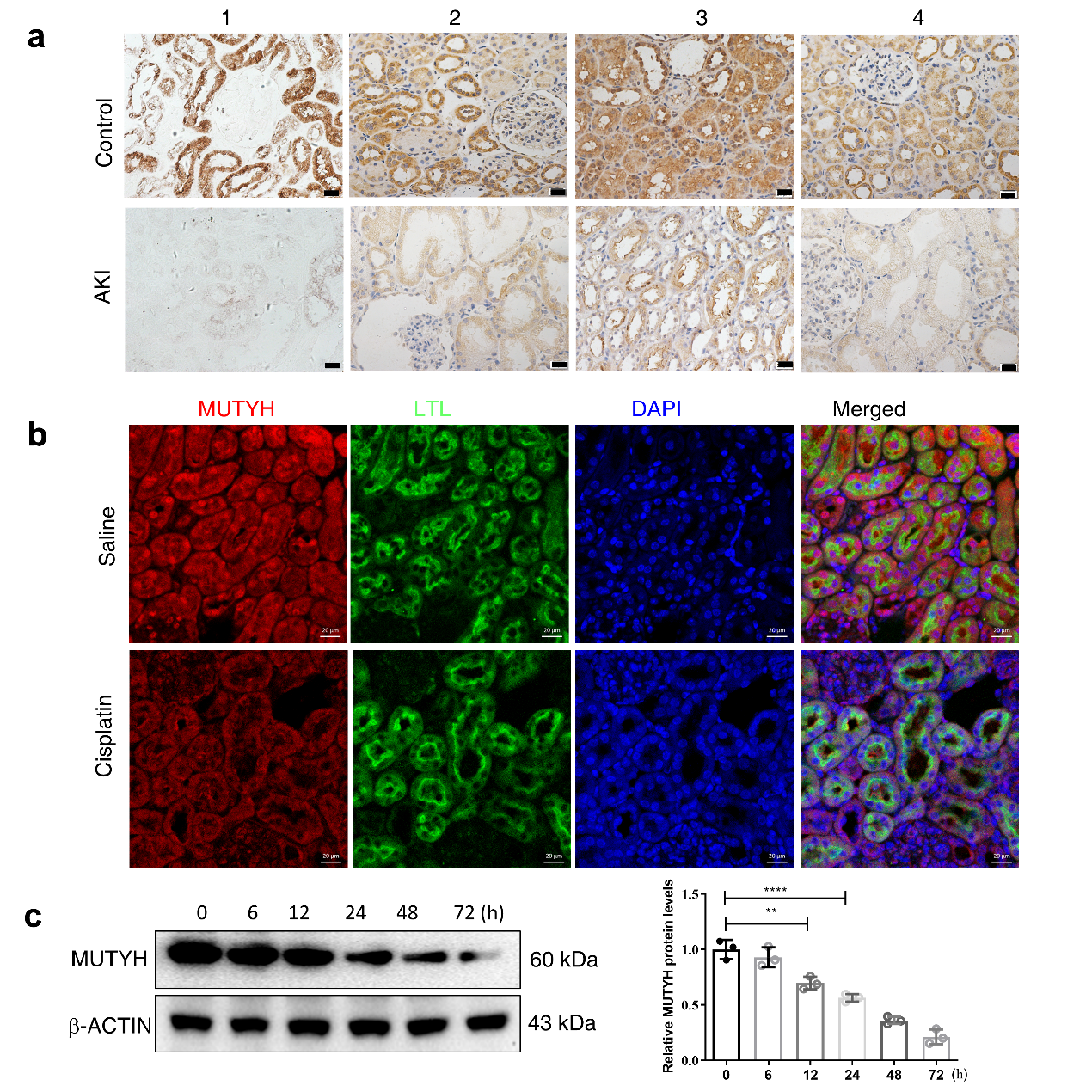


**Figure S1. The experission of MUTYH in kidneys of AKI patients and cisplatin-induced mice.** (**a**) Immunohistochemical staining for MUTYH in the kidneys of patients with AKI (n = 4) and paracarcinoma (n = 4) (magnification: ×400, scale bar: 20 μm). (**b**) Immunofluorescence co-staining of MUTYH with a renal tubule marker, Lotus tetragonolobus lectin (LTL) in kidney tissues of cisplatin-induced (72 h) mice. (**c**) Western blot and quantification of MUTYH protein levels in mice kidneys treated with 20 mg/kg cisplatin for different times. The data are shown as mean ± S.D. for three mice per group (n = 3). ***P<0.01, ****P<0.0001* (one -way ANOVA).

**
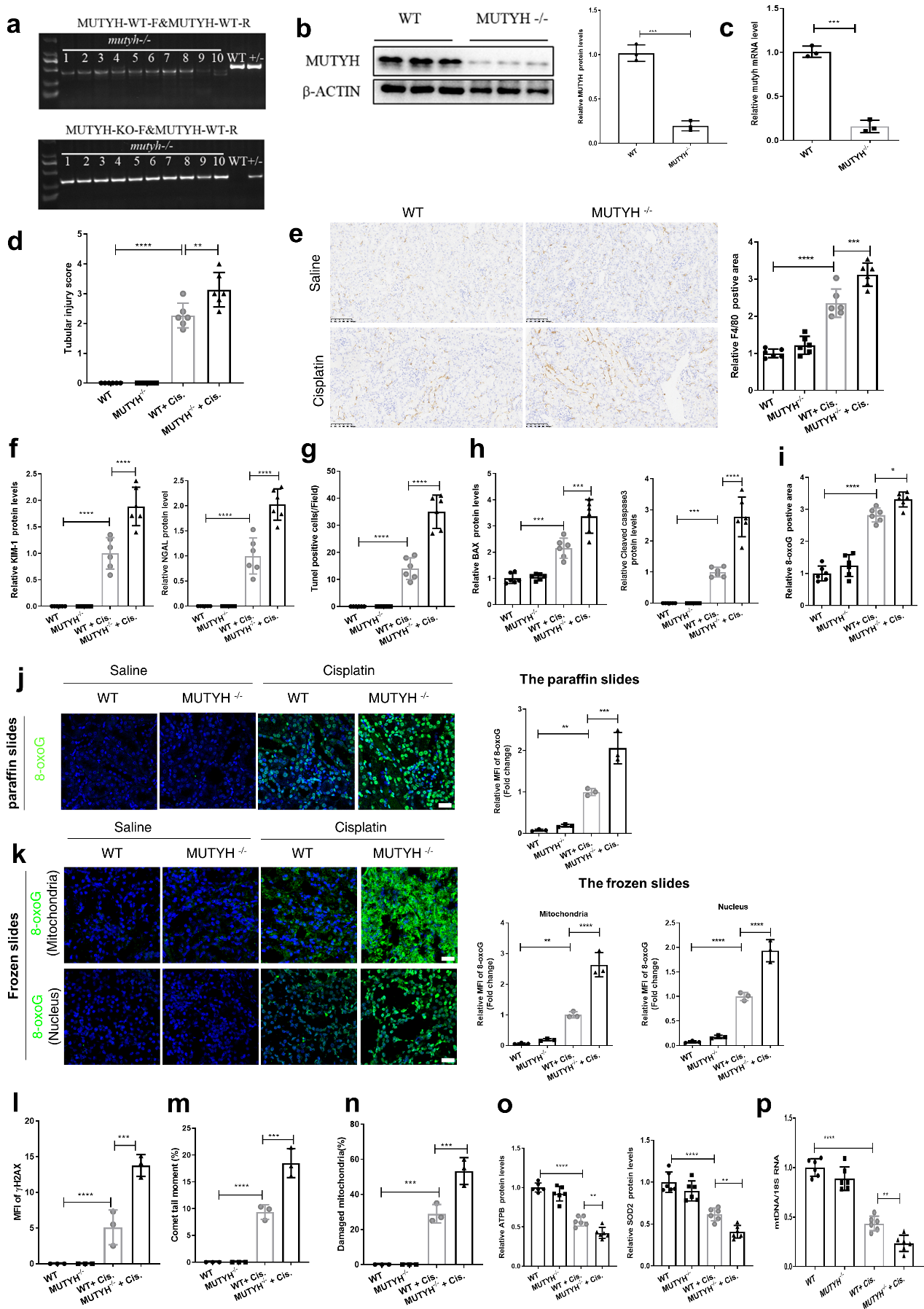
**

**Figure S2. MUTYH knockout aggravates cisplatin-induced AKI.** (**a**) Genotyping results of MUTYH WT, MUTYH^+/-^ and MUTYH^-/-^ mice using a PCR-based method. MUTYH-WT-F, MUTYH-WT-R primer pairs were used to detect WT MUTYH and MUTYH-KO-F, MUTYH-WT-R primer pairs were used to detect mutant alleles. (**b**) Representative western blot analysis of MUTYH in the kidneys of cisplatin-treated WT and MUTYH^-/-^ mice. (**c**) RT-PCR analysis the expression of MUTYH in kidneys of cisplatin-induced WT and MUTYH^-/-^ mice. **(d)** The quantification results of Figure 2b, five random fields were taken from each kidney. (**e**) Immunohistochemical staining for F4/80 in the kidneys of cisplatin-treated (72 h) WT and MUTYH^-/-^ mice (magnification: ×200). Quantification of immunohistochemical staining analyzed by Image J is shown on the right. **(f)** The quantification results of Figure 2d. (**g**) The quantification results of Figure 2e. (**h**) The quantification results of Figure 2f. (**i**) The quantification results of Figure 2G. (**j**) Representative immunofluorescent images of 8-oxoG (N45.1, MOG020P, JaICa) by cisplatin treatment for 72 h in WT or MUTYH^-/-^ mice were obtained using kidney paraffin slides. (**k**) Immunofluorescent images of 8-oxoG (ab62623, Abcam) in mitochondria (top) or nuclei (bottom), by cisplatin treatment for 72 h in WT or MUTYH^-/-^ mice, were acquired using kidney frozen slides. The quantification results are shown on the right. (Scale bar: 20 μm, green: 8-oxoG, blue: DAPI). (**l**) The quantification results of Figure 2h. (**m**) The quantification results of Figure 2i. (**n**) The quantification results of Figure 2j. (**o**) The quantification results of Figure 2k. (**p**) Mitochondria copies number. The data are shown as mean ± S.D. for six or three mice per group (n = 3 or 6). Cis: cisplatin, **P<0.05, **P<0.01, ***P<0.001* (t test or one -way ANOVA).


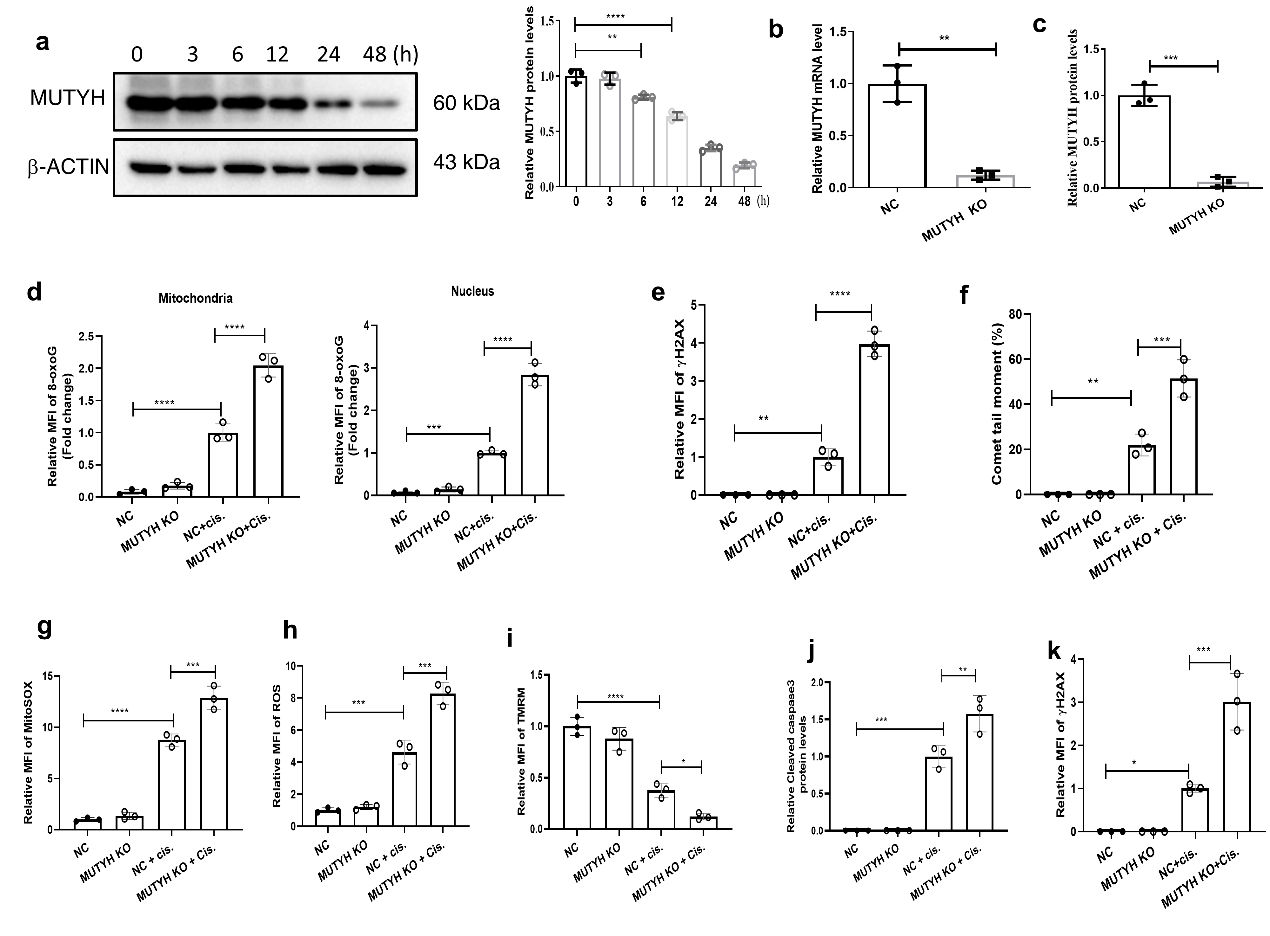


**Figure S3. QRT-PCR were performed to confirm the successful construction of MUTYH^-/-^ mPTCs and the quantification results of Figure 3.** (**a**) Western blot and quantification of MUTYH protein levels in mPTCs treated with 5 μg/ml cisplatin for different times. **(b)** MUTYH sgRNA-1 and MUTYH sgRNA-2 plasmids were co-transfected into mPTCs with Lipofectamine® 2000 and positive cells were selected using puromycin (2 μg/ mL) for three days prior to clonal expansion. QRT-PCR analysis of MUTYH mRNA levels in NC and MUTYH KO mPTCs. (**c**) The quantification results of Figure 3b. (**d**) The quantification results of Figure 3c. (**e**) The quantification results of Figure 3d. (**f**) The quantification results of Figure 3e. (**g**) The quantification results of Figure 3f. (**h**) The levels of ROS in NC and MUTYH KO mPTCs treated with cisplatin was analyzed by FACS. (**i**) The quantification results of Figure 3g. (**j**) The quantification results of Figure 3j. (**k**) The quantification results of Figure 3k. Cell experiments were performed three times, and data are expressed as mean ± S.D (n = 3). NC: negative control, KO: knockout, Cis: cisplatin; *****P<0.001, ***P<0.001, **P<0.01, *P<0.05* (t test or one-way ANOVA).


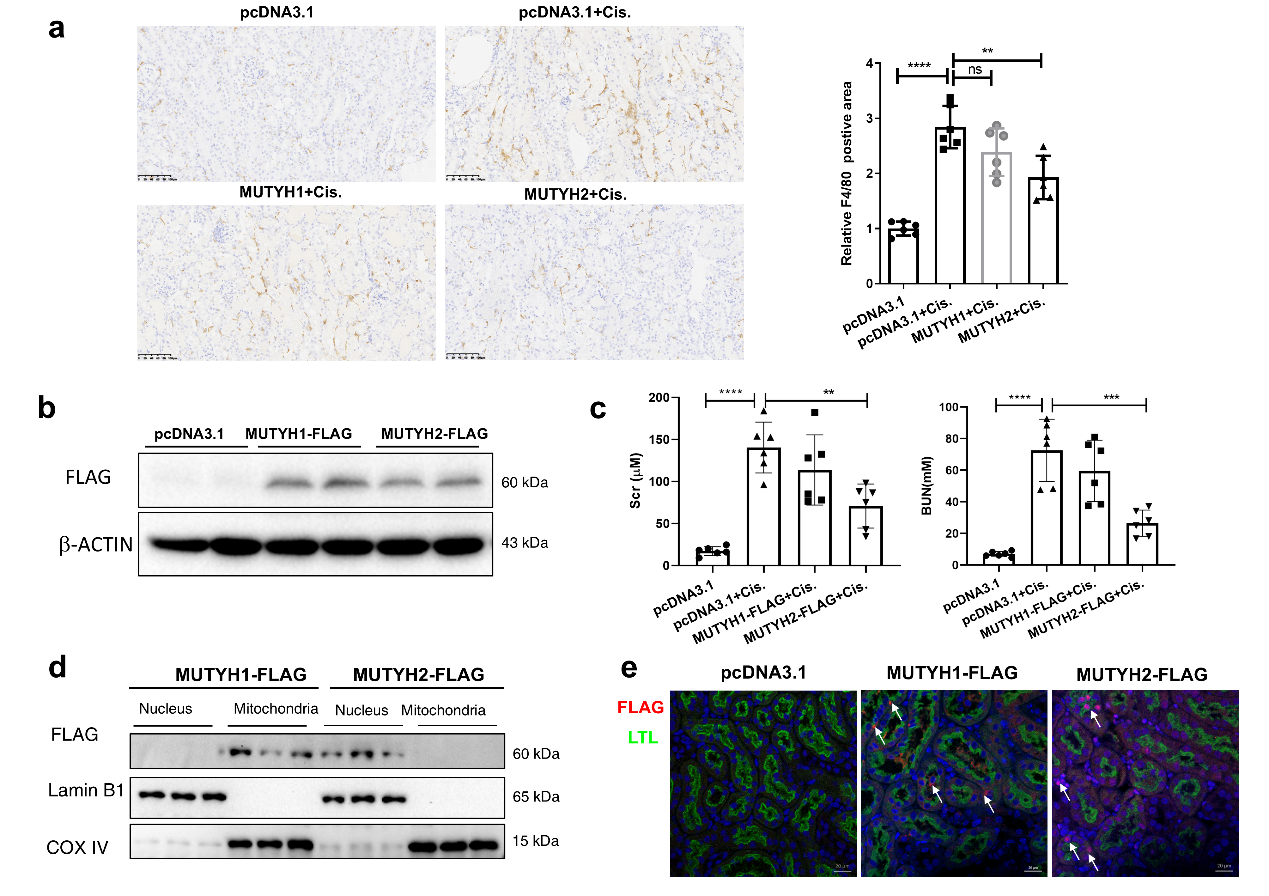


**Figure S4.** **Overexpression of Flag tagged type 2 MUTYH ameliorated cisplatin-induced AKI.** (**a**) Immunohistochemical staining for F4/80 in the kidneys of cisplatin-treated (72 h) with type 1 or 2 human MUTYH overexpression in mice (magnification: ×200). Quantification of immunohistochemical staining analyzed by Image J is shown on the right. (**b**) Western blot analysis of ectopic MUTYH protein levels by FLAG antibody in the kidneys 36 h after injecting FLAG-tagged type 1 or 2 human MUTYH and vector plasmids. (**c**) BUN and Scr levels in control and MUTYH overexpression mice treated with cisplatin for 72 h. (**d**) the nuclear and mitochondrial ectopic MUTYH protein levels in mice kidneys 36 h after injecting FLAG-tagged type 1 or 2 human MUTYH were analyzed by fraction western blot. (**e**) Immunofluorescence staining of ectopic human MUTYH1 and MUTYH2 by using a FLAG antibody in kidneys with injection of vector, human MUTYH1 or 2 overexpression plasmids for 36 h. The results are shown as mean ± S.D. for six mice per group (n = 6). *****P<0.0001, ***P<0.001, **P<0.01* (one-way ANOVA).


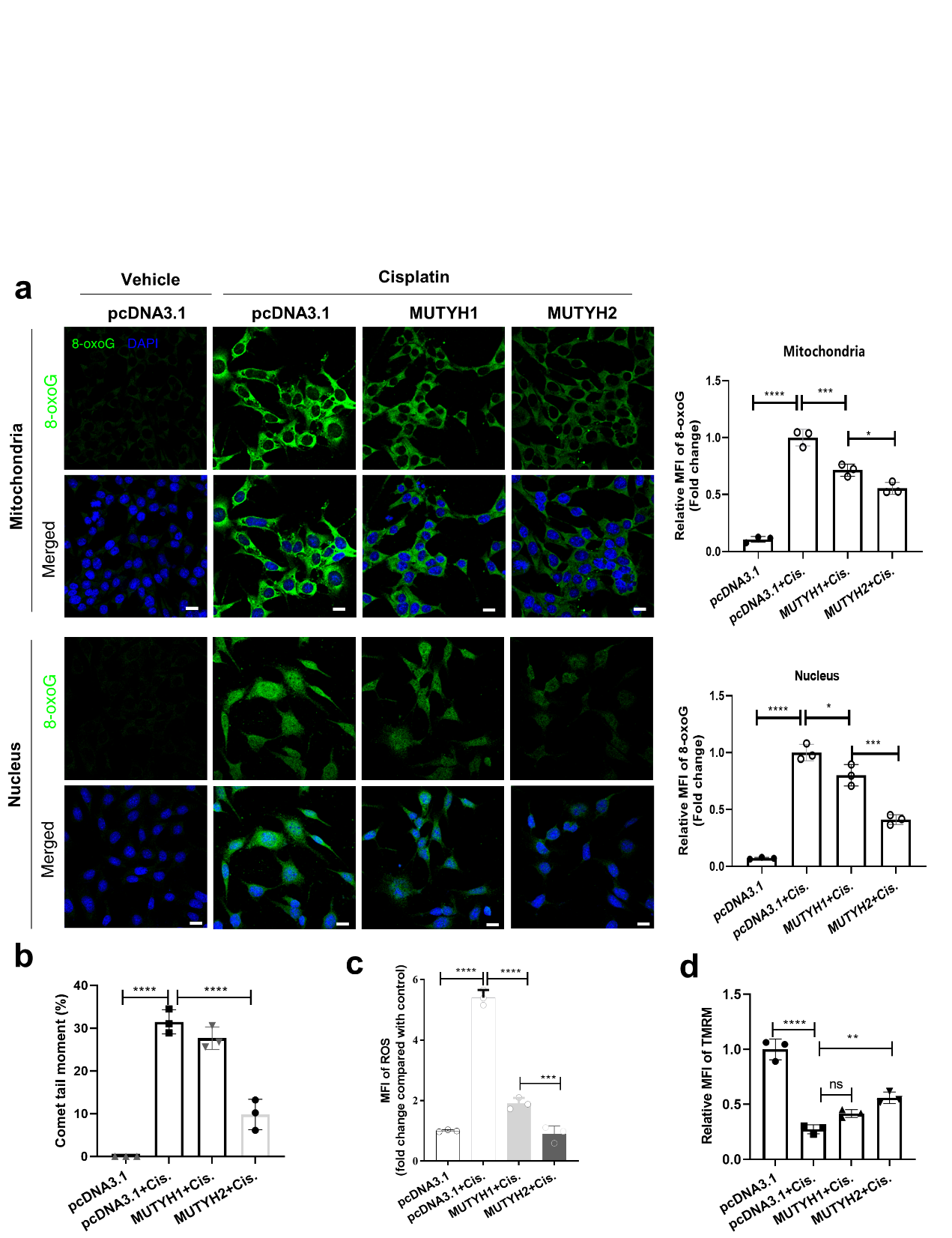


**Figure S5. Immunofluorescent images of 8-oxoG and the quantification results of Figure 5.** (**a**) Immunofluorescent images of 8-oxoG in mitochondria (upper) or nucleus (down) in MUTYH overexpressing mPTCs treated with cisplatin for 24 h (scale bar: 20 μm, green: 8-oxoG, blue: DAPI). The quantification results are shown on the right. (**b**) The quantification results of Figure 5e. (**c**) The levels of ROS in MUTYH overexpressing mPTCs treated with cisplatin was analyzed by FACS. (**d**) The quantification results of Figure 5f. Cell experiments were performed three times, and data are expressed as mean ± S.D (n = 3). Ctrl: control, Cis: cisplatin; ****P<0.001, **P<0.01, *P<0.05* (one-way ANOVA).


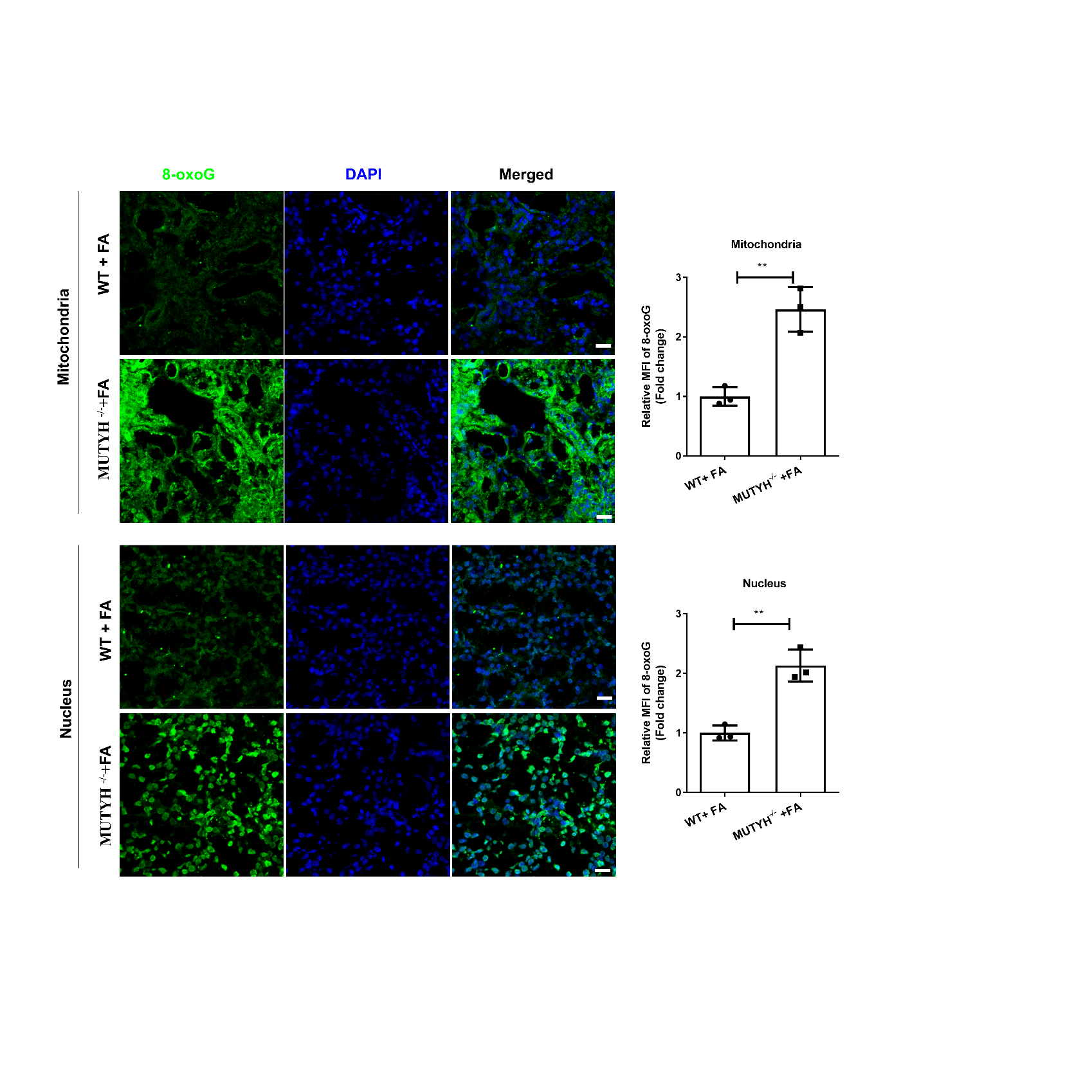


**Figure S6.** Representative immunofluorescent images of 8-oxoG in mitochondria (upper) or nucleus (down) induced by FA (72 h) in kidneys of WT or MUTYH^-/-^ mice (scale bar: 20 μm, green: 8-oxoG, blue: DAPI), the graph shows the quantification results. The results are shown as mean ± S.D. for three mice per group (n = 3). ***P<0.01* (*t-test*).


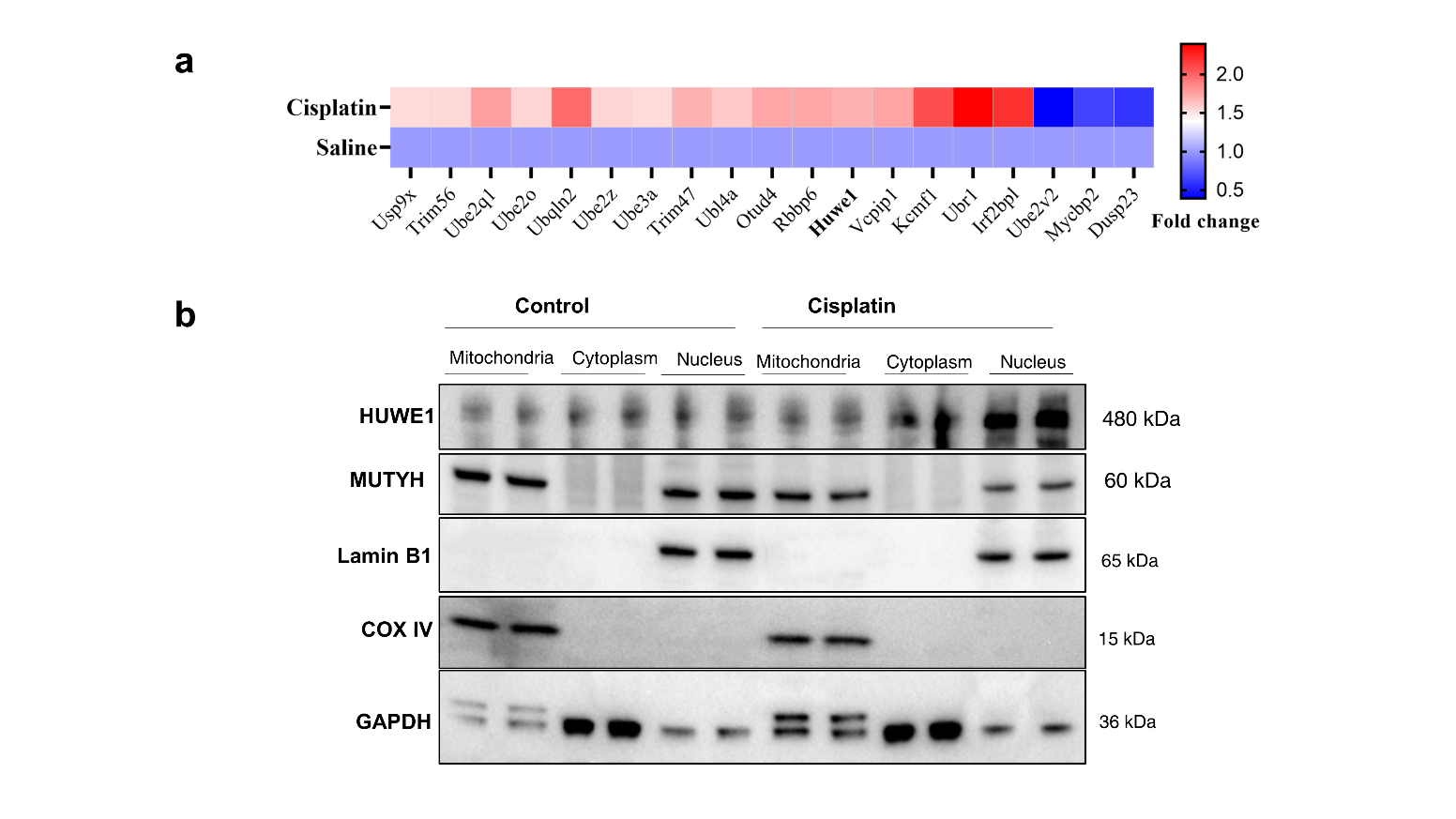


**Figure S7. The expressions of HUWE1 were upregulated in cisplatin-induced mice and mPTCs. (a)** Heat map for differentially expressed ubiquitination and deubiquitinase proteins in the kidneys of saline and cisplatin treatment mice based on Label-free quantification proteomic analysis, one sample of each group. (**b**) The HUWE1 and MUTYH protein levels in nuclear, cytoplasm and mitochondrial of mPTCs treated with 5 μg/ml cisplatin for 24 h were analyzed by fraction Western blot.


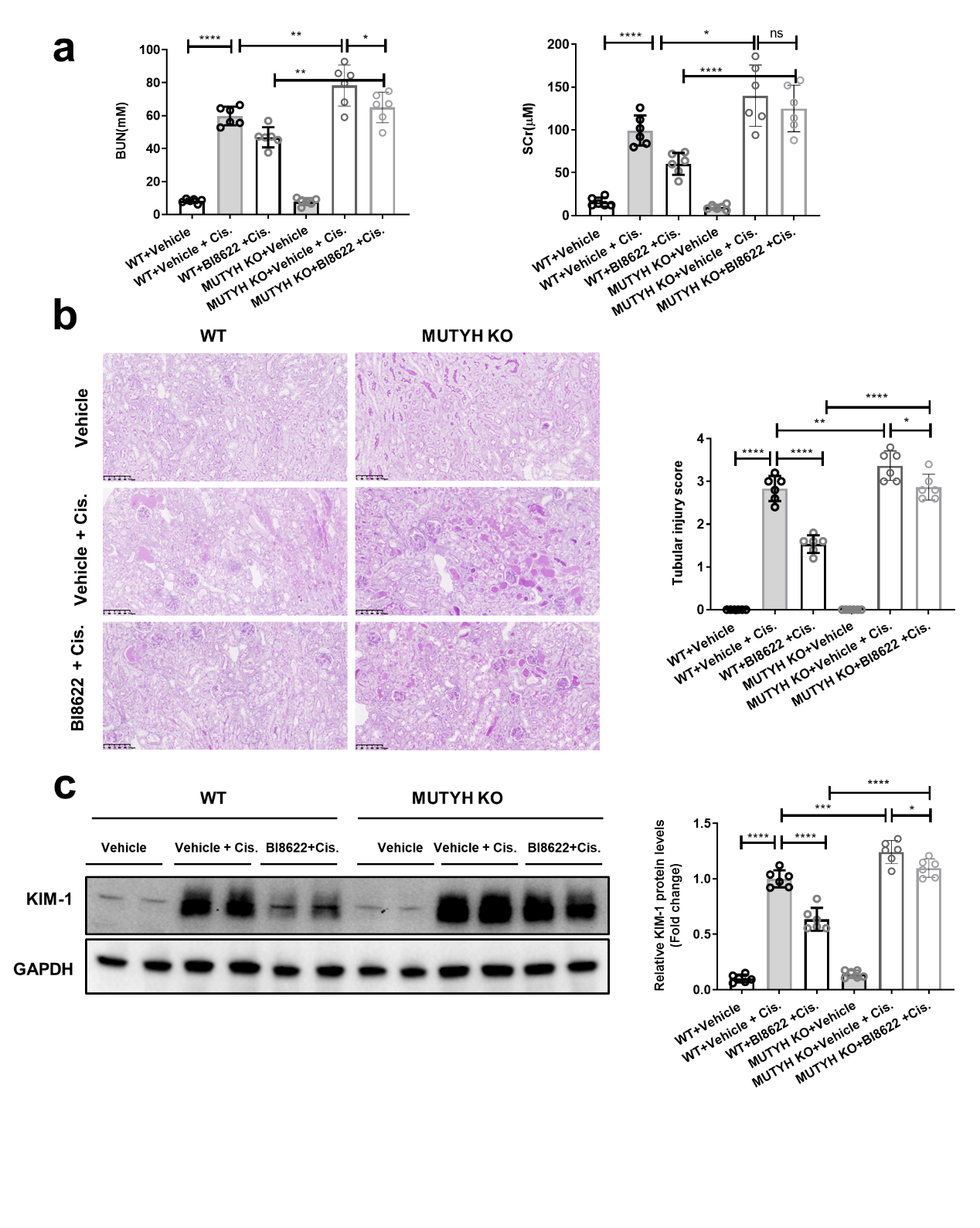


**Figure S8. The effects of BI8622 on MUTYH KO mice.** (**a**) BUN and Scr levels in WT or MUTYH KO mice after i.p. injection of BI8622 and 72 h cisplatin treatment. (**b**) PAS staining images (magnification: ×200) of kidneys from WT or MUTYH KO mice after i.p. injection of BI8622 and 72 h cisplatin treatment and tubular injury scores (right). Five random fields were taken from each kidney. (**c**) Western blot analysis of KIM-1 protein levels in the kidneys of WT or MUTYH KO mice after i.p. injection of BI8622 and 72 h cisplatin treatment. The data was expressed as means ± S.D. n = 6, ns: no significant; *****P<0.0001, **P<0.01, *P < 0.05 (one way ANOVA)*.

**
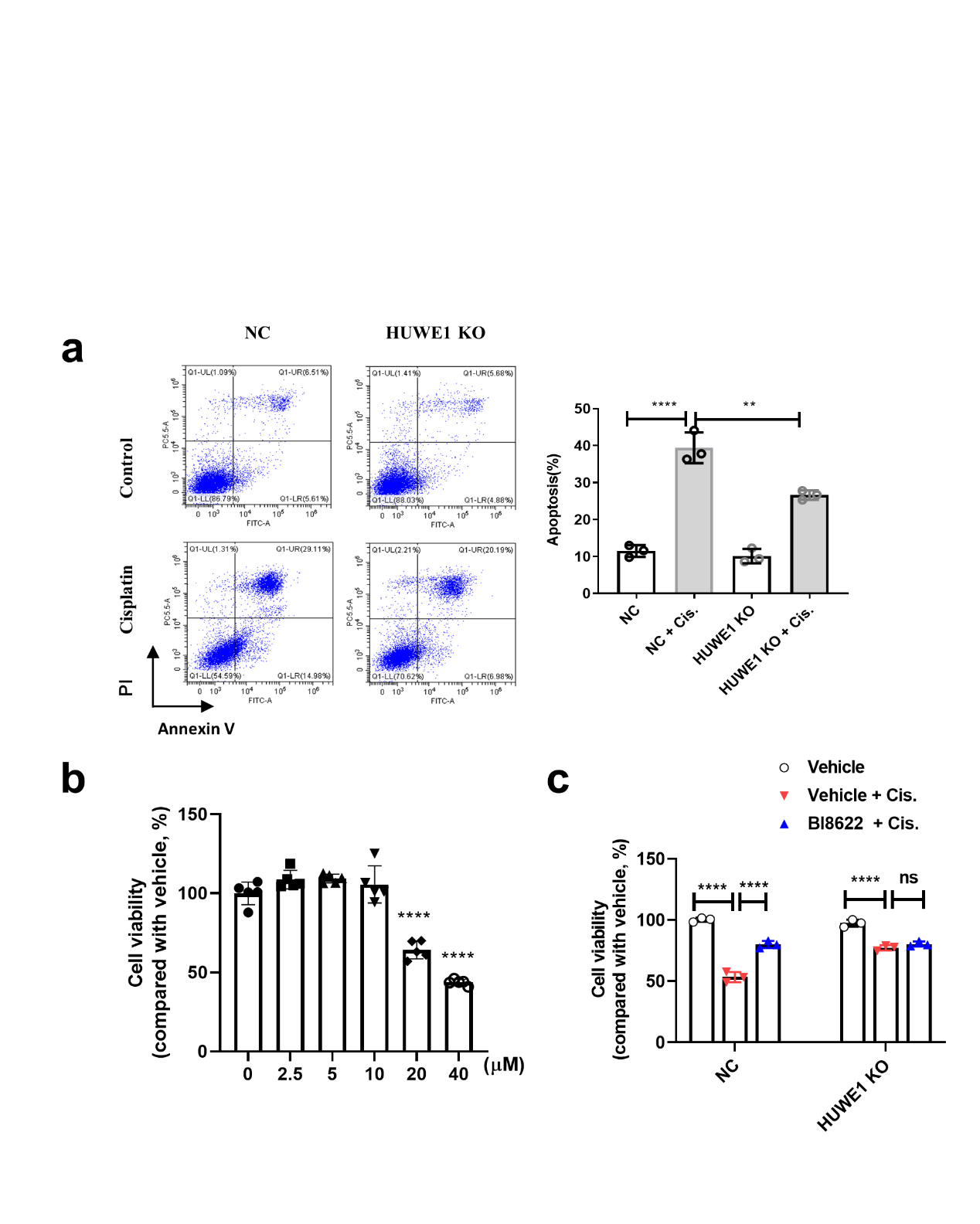
**

**Figure S9. Inhibition or knockout of HUWE1 ameliorated cisplatin-induced injury in mPTCs.** (**a**) Flow cytometry analysis of Annexin V and PI staining and quantification of apoptosis induced by cisplatin in HUWE1 knockout mPTCs. (**b**) CCK8 assay was performed to analyze the cell viability of mPTCs after treatment with different concentration of BI8622 for 24 h. (**c**) CCK8 assay was performed to analyze the protect effects of BI8622 (10 μM) on HUWE1 knockout and control mPTCs treatment with cisplatin. Cell experiments were performed for three times and the data was expressed as means ± S.D (n = 3 or 5). NC: Negative control, Cis. Cisplatin, ns: no significant; ****P<0.001, **P<0.01, *P < 0.05 (one or two-way ANOVA)*.

**Table S1. Clinical data of AKI patients**

| Number | SCr(μM) | BUN (mM) | Age & sex | cause of AKI |
| --- | --- | --- | --- | --- |
| 3139 | 91 | - | 13 years old female | Drug and infection |
| 3188 | 618 | 34.50 | 12 years old male | Drug and infection |
| 2017-3226 | 476 | 18.62 | 6 years old female | Infection |
| 2017-3227 | 548 | 18.73 | 1 year old female | Acute interstitial nephritis |

**Table S2. Clinical data of renal tumor patients**

| Number | SCr(μM) | BUN (mM) | Age & sex | Tumor |
| --- | --- | --- | --- | --- |
| 201900811 | 31 | 3.5 | 4 years old male | nephroblastoma |
| 202316192 | 13.9 | 1.70 | 1 month old male | nephroblastoma |
| 202316207 | 25.3 | 3.01 | 6 months old male | nephroblastoma |
| 202308593 | 33 | 3.6 | 3 years old male | nephroblastoma |

**Table S3. Primer Sequences**

| Gene | Primer Sequence (5’-3’) |
| --- | --- |
| Mouse IL-6 | F: ACAAAGCCAGAGTCCTTCAGAGAG  R: TTGGATGGTCTTGGTCCTTAGCCA |
|  |  |
| Mouse TNF-α | F: TCCCCAAAGGGATGAGAAG  R: CACTTGGTGGTTTGCTACGA |
|  |  |
| Mouse MUTYH | F: TGCCACCTGTGTTGTGGAGC  R: TGTGCTGATGCTGTTCTGAGGG |
|  |  |
| Mouse mt-ND1 | F: ACACTTATTACAACCCAAGAACACAT  R: TCATATTATGGCTATGGGTCAGG |
|  |  |
| Mouse mt-ATP8 | F: ACATTCCCACTGGCACC  R: GGGGTAATGAATGAGGC |
| Mouse mt-COX1 | F: CAGACCGCAACCTAAACACA  R: TTCTGGGTGCCCAAAGAAT |
| Mouse mt-COX2 | F: GCCGACTAAATCAAGCAACA  R: CAATGGGCATAAAGCTATGG |
| Mouse mt-ATP6 | F: CCATAAATCTAAGTATAGCCATTCCAC  R: AGCTTTTTAGTTTGTGTCGGAAG |
| Mouse mt-cytb | F: TTCTGAGGTGCCACAGTTATT  R: GAAGGAAAGGTATTAGGGCTAAA |
| Mouse β-actin | F: GAGACCTTCAACACCCCAGC  R: ATGTCACGCACGATTTCCC |
|  |  |
| mtDNA | F: ATCCTCCCAGGATTTGGAAT  R: ACCGGTAGGAATTGCGATAA |
| 18S rRNA | F: TTCGGAACTGAGGCCATGATT  R: TTTCGCTCTGGTCCGTCTTG |
| MUTYH-WT-F | TGCCACCTGTGTTGTGGAGC |
| MUTYH-WT-R | GCAGTAGACACAGCTGCAT |
| MUTYH-KO-F | CTACGCATCGGTAATGAAGG |
| Mouse MUTYH sgRNA-1 | F: CACCGATATCATCTCTTCAGCGACG  R: AAACCGTCGCTGAAGAGATGATATC |
| Mouse MUTYH sgRNA-2 | F: CACCGCCCGGCATAAACTCTGCACG  R: AAACCGTGCAGAGTTTATGCCGGGC |
| Mouse HUWE1 sgRNA-1 | F: CACCGATCTGTTGGACCGCTTTGA  R: AAACTCAAAGCGGTCCAACAGATC |
| Mouse HUWE1 sgRNA-2 | F: CACCGTGATTTGCTGCAGTTCCAAG  R: AAACCTTGGAACTGCAGCAAATCAC |
| SAM system primers of mouse HUWE1 sgRNA-1 | F: CACCGAGGCGCGGGCGGTCTCCTG  R: AAACCAGGAGACCGCCCGCGCCTC |
| SAM system primers of mouse HUWE1 sgRNA-2 | F: CACCGTGGTAAGCGAGATCCCTAA  R: AAACTTAGGGATCTCGCTTACCAC |
